# Multilayered Fabrication Containing Wind Turbine Blade Solid Wastes for High-Performance Composite Fibers

**DOI:** 10.1021/acsmaterialsau.5c00041

**Published:** 2025-07-17

**Authors:** Varunkumar Thippanna, Arunachalam Ramanathan, Dhanush Patil, M. Taylor Sobczak, Taylor G. Theobald, Sri Vaishnavi Thummalapalli, Xiao Sun, Churan Yu, Ian Doran, Chao Sui, Joshua Were, Xianqiao Wang, Sui Yang, Xin Xu, Arunachala Nadar Mada Kannan, Amir Asadi, Ayman Nafady, Abdullah M. Al-Enizi, Mohammad K. Hassan, Kenan Song

**Affiliations:** † Mechanical Engineering, College of Engineering, 1355 University of Georgia, 302 E Campus Rd, Athens, Georgia 30602, United States; ‡ Department of Mechanical and Industrial Engineering, College of Engineering, Northeastern University, 360 Huntington Avenue, Boston, Massachusetts 02115, United States; § School of ECAM, College of Engineering, University of Georgia, 302 E Campus Rd, Athens, Georgia 30602, United States; ∥ Materials Science and Engineering, School for Engineering of Matter, Transport and Energy (SEMTE), Ira A. Fulton Schools of Engineering, 7864 Arizona State University, Tempe, Arizona 85281, United States; ⊥ The Polytechnic School (TPS), Ira Fulton Schools of Engineering, Arizona State University, Mesa, Arizona 85212, United States; # Manufacturing and Mechanical Engineering Technology, Department of Engineering Technology and Industrial Distribution, 14736 Texas A&M University, College Station, Texas 77843-3367, United States; ¶ Department of Chemistry, College of Science, 37850 King Saud University, Riyadh 11451, Saudi Arabia; ∇ Center for Advanced Materials, 61780 Qatar University, Doha 2713, Qatar; ○ Department of Mechanical Engineering, College of Engineering, University of Georgia (UGA), 302 E. Campus Rd, Athens, Georgia 30602, United States

**Keywords:** wind turbine blades, multilayered fibers, heat treatment, carbonized fibers, thermal stability

## Abstract

The disposal of wind turbine blade (WTB) waste poses a significant environmental challenge due to its high volume and complex composition. This study introduces an innovative approach to address this issue by repurposing WTB-derived glass fibers (GF) into high-performance polyacrylonitrile (PAN)-GF composite fibers through a scalable dry-jet wet spinning and forced assembly process. By integrating alternating layers of PAN and PAN-GF, layer thickness was precisely controlled to the micrometer scale, ensuring enhanced GF dispersion and improved orientation through shear stress at layer interfaces. The individual layer thickness in the multilayered PAN-GF fibers decreased progressively with an increasing number of layers, with 32-layered fibers exhibiting comparatively thicker layers, while 256-layered fibers demonstrated significantly thinner layers. The effects of WTB-GF incorporation on the thermal and mechanical properties of PAN fibers were examined using tensile testing and thermogravimetric analysis (TGA). Using GF loadings of 1–4 wt %, the 256-layered composite fibers demonstrated remarkable mechanical improvements, with stiffness (modulus) increasing by 54.7% from 15.10 to 23.37 GPa and tensile strength rising by 27.2% from 521.71 to 663.66 MPa compared to pure PAN fibers. TGA results indicate that increasing the GF content leads to higher residual weight at 900 °C, reflecting enhanced thermal stability and greater char yield. The 256-layered 10PAN-4GF fibers showed the highest residual mass (41.23 wt %), highlighting the significant contribution of GF reinforcement to thermal stabilization. Heat treatment further transformed these precursor fibers into carbonized fibers (CF) with exceptional thermal stability and performance under extreme conditions. This process highlights a sustainable pathway for reusing WTB waste and producing advanced composite fibers, making them ideal candidates for demanding applications such as aerospace and space exploration.

## Graphical abstract

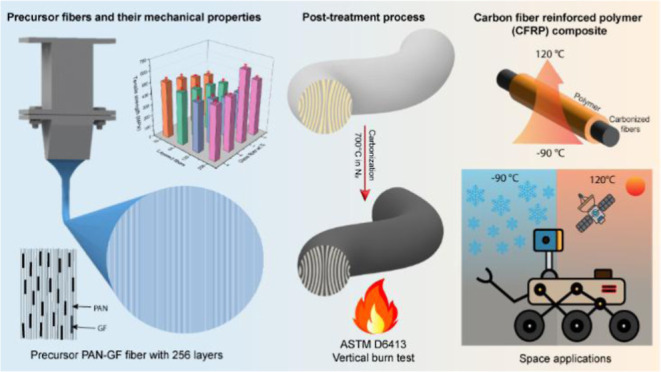

## 1 Introduction

Global plastic consumption now exceeds 380 million tons annually, presenting significant environmental challenges if not properly managed.^1^ Plastics are used extensively across consumer goods and industrial applications, leading to a diverse range of polymers in waste streams.^2^ Thermoplastics, some made of linear polymer chains, can be reshaped with heat, while thermosets, commonly found in industrial products, are cross-linked and retain their shape regardless of temperature changes, making them particularly difficult to recycle.^3^ Improper disposal of plastic waste, especially in landfills, can lead to severe soil and water contamination, posing risks to ecosystems and human health.^4^ Recycling plays a crucial role in reducing energy consumption and mitigating environmental impacts.^5^ However, landfill disposal, while preferable to incineration, is increasingly restricted by legislation prioritizing recycling and the reuse of waste as a valuable resource.^6^ Transforming solid waste into high-value carbon materials is essential for advancing a circular economy, promoting sustainable resource utilization, and minimizing environmental harm.^7–9^

Glass fiber-reinforced polymer (GFRP) composites, such as those used in wind turbine blades (WTBs), present a significant recycling challenge due to their intricate composition and the inherent properties of their thermoset matrices.^10,11^ As a result, most WTBs are discarded in landfills, where they persist for decades, contributing to soil and water contamination and exacerbating environmental degradation.^12,13^ This issue is particularly pressing, as global wind turbine deployment continues to expand, producing thousands of tons of end-of-life WTB waste annually, with projections indicating nearly 8000 tons of blade mass requiring disposal in regions like Maine alone by 2035.^14,15^ Current recycling methods for GFRP composites, including chemical processes, pyrolysis, and mechanical crushing, are largely focused on recovering fibers while neglecting the potential reuse of the entire material.^16–18^ These methods often produce inconsistent fiber quality and suffer from high energy demands, further limiting their viability.^19^ Mitigating these inefficiencies is essential for minimizing the environmental impact of wind energy systems, particularly as the industry transitions to a circular economy.^20,21^ Developing innovative recycling technologies and optimizing glass fibers (GF) reuse could not only minimize environmental pollution but also enhance the sustainability and economic feasibility of composite materials in high-performance applications.^22^ The inclusion of recycled solid waste (such as GF) as filler reinforcement is significantly limited in composite manufacturing scalability, typically restricted to less than 10%.^23^

Polyacrylonitrile (PAN) is the leading precursor for manufacturing high-performance carbon fibers due to its high carbon yield and ability to produce fibers with exceptional properties.^24^ Compared to other precursors like pitch and cellulose, PAN-based carbon fibers excel in mechanical strength,^25^ flame retardancy,^26^ thermal stability,^27^ and resistance to chemical and environmental degradation,^28^ making them the preferred choice for advanced applications. The production of PAN-based carbon fibers typically involves the dry-jet spinning of polymer fibers, followed by thermal processes such as stabilization, carbonization, and graphitization.^29,30^ During stabilization, the chemical structure of the fibers is altered in an atmospheric environment to make them thermally stable and prevent remelting.^31^ Low-temperature carbonization of PAN fibers offers distinct advantages, including the preservation of functional groups like nitrogen and oxygen, which enhance flame retardancy and improve fiber–matrix adhesion.^32,33^ This approach sometimes relies on the template fillers (e.g., GF or nanoparticles) can also minimize the risk of excessive shrinkage or structural degradation,^34^ which are more common at higher carbonization temperatures, thereby preserving the fiber’s structural integrity.^35,36^ Additionally, fibers produced under low-temperature carbonization exhibit reduced brittleness and improved flexibility, making them ideal for applications that require both mechanical resilience^37^ and enhanced functional properties.^38^ This balance of structural transformation and functional retention underscores PAN’s prominence as the preferred precursor for high-performance carbon fiber manufacturing.^37,39^

In this study, we introduce a novel methodology that combines dry-jet wet spinning and layer-by-layer assembly process for the scalable fabrication of multilayered fiber composites, featuring alternating layers of PAN and solid waste from wind turbine blades containing GF. The alternating PAN and PAN-GF layers allow precise control over layer thickness and promote improved fiber dispersion and alignment through interfacial shear. As the number of layers increases, individual layers become progressively thinner, enhancing structural uniformity. The incorporation of recycled glass fibers significantly improves the thermal and mechanical performance of the composite fibers, as confirmed by tensile and thermogravimetric analyses. Notably, higher GF content leads to improved stiffness, strength, and thermal stability, with enhanced char retention at elevated temperatures. Further optimization through heat treatment, the resulting carbonized fibers exhibits excellent durability, positioning them as promising candidates for high-performance applications while supporting sustainable reuse of composite waste.

## 2 Results and Discussion

### 2.1 Overview of the Fiber Processing

The multimaterial layering process begins with two precursor feedstocks that enter a custom-designed multilayered spinneret. Feedstock A contains 10 wt % PAN polymer, while feedstock B comprises PAN-GF with 1 wt % to 4 wt % GF relative to the PAN content. They undergo vertical and horizontal rearrangement as well as splitting, converting two layers into four alternating ones (Figure 1a). The number of layers doubles with each subsequent multiplier, and their thickness is halved. Employing “*n*” multipliers produce 2^*n*+1^ layers.^40–42^ Using 7 multipliers, a PAN-GF consisting of 256 layered fibers is produced (Figure 1b). This process generates high shear forces, aligning alternating PAN macro-molecules more parallel to the fiber axis, thereby significantly enhancing structural order and orientation.^43,44^ GFs are highly oriented due to shear stress developed within the alternative PAN layers, resulting in better alignment of GF along the fiber axis. The SEM image provides a clear representation of the GF oriented along the fiber axis within 256-layered fibers (Figure 1b_1_). However, the GF alignment decreases in fibers with fewer layers, such as 32, 64, and 128 layers (Figure 1c). These highly drawn fibers show that with the increase in layer numbers, the orientation of GF improves and thus improves the properties of the fibers. The thickness of the as-spun fiber is primarily influenced by the speed of the collection winder and the flow rate of the polymer solution during spinning, the final fiber diameter depends on the initially collected as-spun fibers (Table S1). The stretchability of the as-spun fibers is significantly impacted by stretching in the baths (water and oil) at different temperatures, to achieve a high degree of polymer/filler orientation and crystallinity, thereby enhancing mechanical properties.^45^

**Figure 1:**
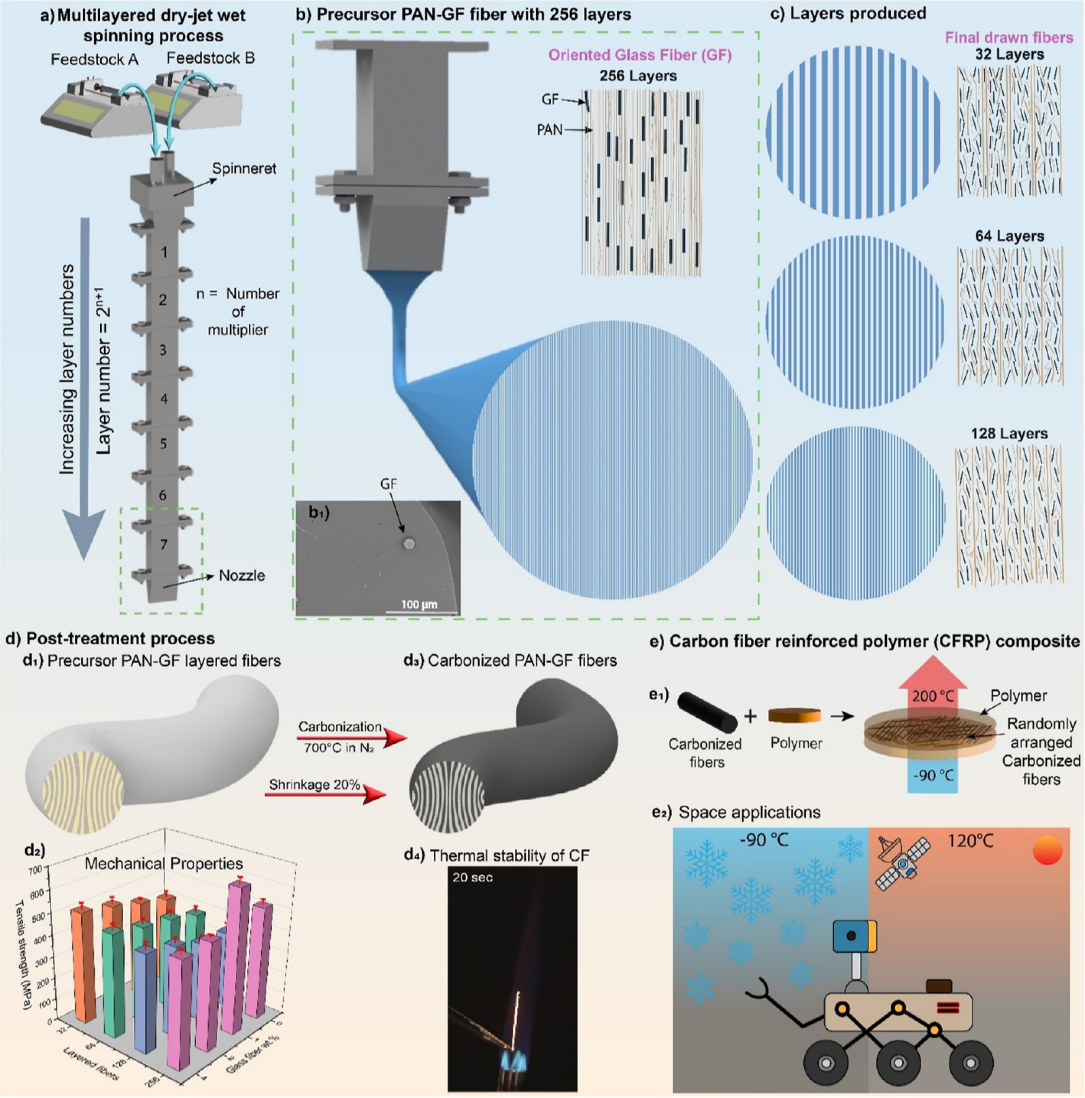
Schematic overview of the PAN-GF composite fibers with multilayered structures and optimized properties. (a) Multilayered dry-jet wet spinning process of two feedstocks with increasing layer numbers to produce (b) PAN-GF composite fibers with 256 layers, with (b_1_) oriented GF protruding along the fiber axis. This is in contrast with (c) other PAN-GF composite fibers with 32, 64, and 128 layers with preferential GF orientation, less aligned along the fiber axis. (d) Post-treatment process for the composite fibers with (d_1_) the highest drawn precursor PAN-GF fibers showing multilayered microstructure, (d_2_) their mechanical properties (i.e., tensile strength), as a function of different layer numbers and varying GF concentrations (wt %), and (d_3_) the precursor fibers after the heat-treatment process to produce carbonized fibers (i.e., with a 20% shrinkage at the 700 °C carbonization), as well as (d_4_) the vertical burn test showing the thermal stability of the carbonized fibers. (e) Carbon fiber-reinforced polymer (CFRP) composite uses.

During the postprocessing, these fibers were subjected to a drawing process at 85 °C in water to remove residual solvent from the fibers. Washing in water was performed simultaneously with the fiber stretching process, followed by additional stretching at 145 °C in silicone oil to produce high-draw-ratio dense fibers (Figure S1) with a layered structure and fewer voids (Figure 1d_1_). During the bath drawing process, fibers were stretched continuously before they reached their breaking point. The fibers subjected to higher draw ratio (DR) resulted in increased strength due to enhanced molecular alignment and compresses internal pores, all essential processes for creating high-strength fibers. Following this, the high DR fibers produced via winders underwent manual stretching on a hot plate above the glass transition, resulting in pore collapse to maximize their tensile properties (e.g., strength); also, the 256-layered fibers containing varying GF wt % with the maximum strength (Figure 1d_2_). This final stretching step led to a fiber thickness of 50–60 μm.

Besides, these precursor fibers underwent a heat-treatment process that includes stabilization in the air atmosphere at a moderate temperature of 260 °C and carbonization at 700 °C in an inert atmosphere to produce CF, which also generated a 20% shrinkage as compared to the precursor fibers (Figure 1d_3_). The heat treatment process altered the fiber structure, removed mostly hydrogen elements, and formed strong, high-performance CF with improved thermal properties capable of withstanding a high temperature (Figure 1d_4_). Carbon fiber-reinforced polymer (CFRP) composites can be made by embedding CF within a polymer matrix, creating a durable material capable of surviving extreme environments (Figure 1e_1_). Due to their lightweight, high strength, and thermal stability, these fibers are increasingly used in space applications (Figure 1e_2_).

### 2.2 Mechanical Performance

#### 2.2.1 Fiber Diameter Influences

The fiber diameter plays a critical role in determining the mechanical properties of fibers, as shown in Table 1. Reducing the fiber diameter minimizes defect density, contributing to improved strength and stiffness.^46^ For example, fibers with thinner diameters, such as the 10PAN-1GF fibers at 256 layers (52 μm), exhibit a high tensile strength of 663.66 ± 28.67 MPa and a Young’s modulus of 23.37 ± 3.97 GPa. This relationship aligns with the Griffith hypothesis, where smaller diameters reduce flaws, enabling fibers to approach their theoretical maximum strength and modulus.^47^ The combination of reduced diameter and GF reinforcement further enhances the matrix’s mechanical properties by promoting strong matrix–fiber bonding and effective load transfer. However, achieving this performance requires precise control over GF concentration and distribution to prevent agglomeration, which can introduce defects and diminish composite performance. For instance, at higher GF concentrations (10PAN-4GF), there is a slight reduction in tensile strength compared to 10PAN-1GF, despite similar diameters, emphasizing the importance of uniform dispersion and optimal GF content for maximizing fiber properties.

**Table 1:** Mechanical Properties of the Precursor Layered Fibers and Their Composition with the Layer Numbers

		mechanical properties of prestabilized fibers			
layers	fiber type	Youngs modulus (GPa)	tensile strength (MPa)	elongation at break (%)	diameter of manually stretched fibers (μm)
32	10PAN	9.96 ± 0.65	412.19 ± 28.09	12.31 ± 2.65	58
	10PAN-1GF	12.60 ± 2.01	446.76 ± 09.24	12.28 ± 2.42	60
	10PAN-2GF	12.43 ± 2.04	485.41 ± 29.98	13.35 ± 2.60	61
	10PAN-4GF	14.69 ± 3.74	513.23 ± 29.19	8.64 ± 2.58	58
64	10PAN	9.15 ± 1.74	386.08 ± 28.78	9.82 ± 2.99	60
	10PAN-1GF	10.01 ± 0.37	422.17 ± 26.73	11.79 ± 2.88	55
	10PAN-2GF	10.05 ± 0.53	444.32 ± 26.46	12.57 ± 1.49	61
	10PAN-4GF	10.26 ± 0.90	475.09 ± 28.99	16.50 ± 1.29	50
128	10PAN	12.35 ± 0.92	350.20 ± 35.51	9.81 ± 2.01	51
	10PAN-1GF	8.95 ± 1.17	363.05 ± 23.71	10.98 ± 0.55	51
	10PAN-2GF	10.42 ± 1.06	415.36 ± 26.27	9.56 ± 3.70	55
	10PAN-4GF	16.93 ± 2.65	449.17 ± 31.87	8.64 ± 1.39	57
256	10PAN	15.10 ± 1.07	521.71 ± 25.05	10.21 ± 1.74	51
	10PAN-1GF	23.37 ± 3.97	663.66 ± 28.67	9.69 ± 3.05	52
	10PAN-2GF	16.82 ± 3.78	493.31 ± 13.20	10.34 ± 1.63	51
	10PAN-4GF	14.24 ± 2.73	486.25 ± 22.51	11.52 ± 3.79	53

#### 2.2.2 Reinforcement of Filler Impacts

The tensile properties of the manually stretched fibers are summarized in Table 1. The inclusion of GF derived from solid waste, such as wind turbine blades, has proven to be an effective strategy for reinforcing polymer composites. GF acts as a mechanical filler, significantly enhancing the stiffness and strength of the composite by distributing stress and reducing localized failure points. For example, in 32-layered fibers (Figure 2a_1_), incorporating 1 wt % GF into the PAN matrix increased the tensile strength from 412.19 MPa (pure PAN) to 446.76 MPa, and further to 513.23 MPa at 4 wt % GF. Similarly, the modulus improved from 9.96 GPa in pure PAN to 14.69 GPa for 10PAN-4GF. These improvements underscore the effectiveness of GF as reinforcement, enabling the composite to bear higher loads and maintain structural integrity under stress. This demonstrates that recycled solid waste from wind turbine blades, with their inherent GF composition, can serve as efficient mechanical reinforcement fillers, offering a sustainable solution for repurposing waste into high-performance materials.

**Figure 2:**
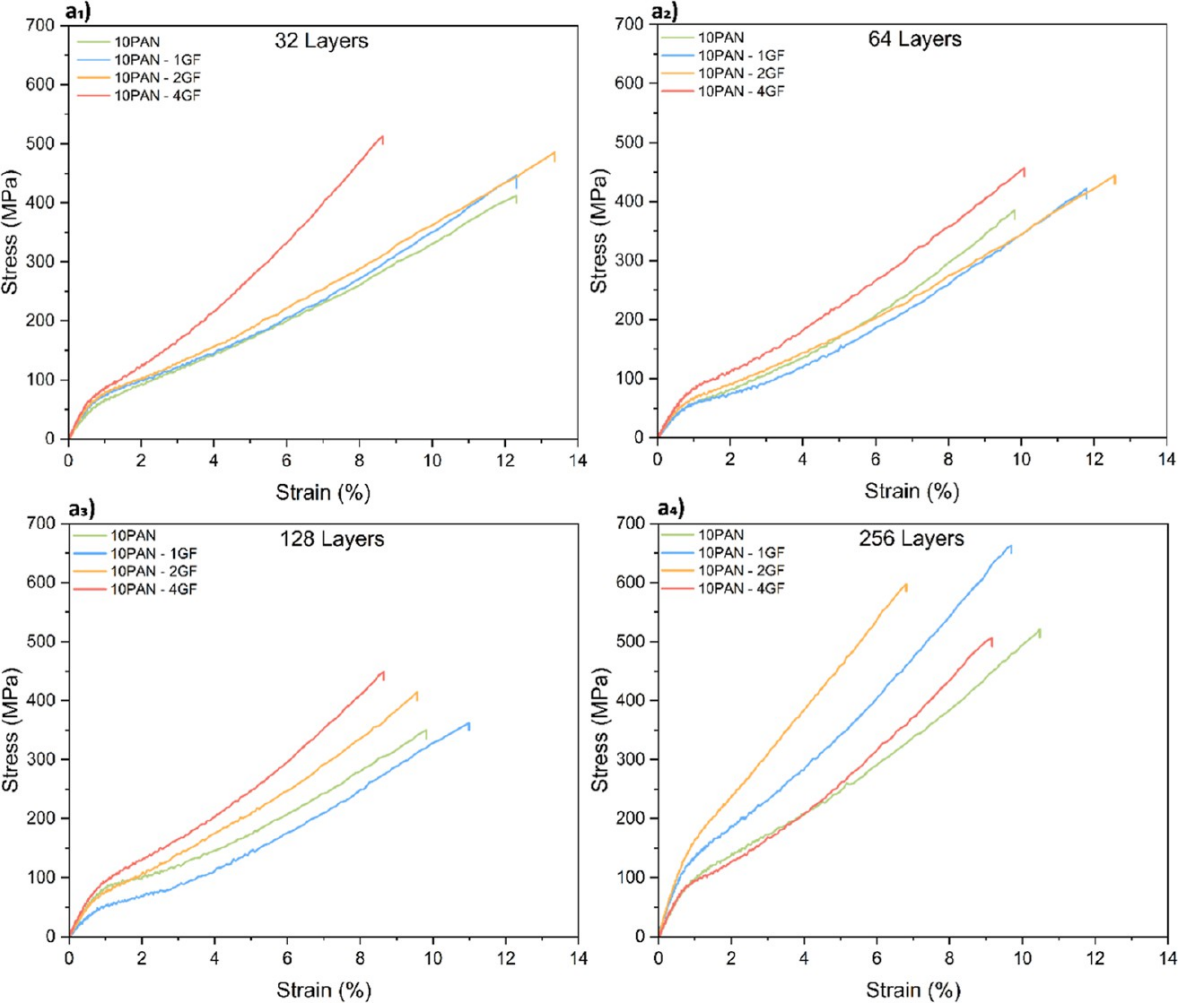
Mechanical properties of the precursor fibers with different layers. (a_1_) Stress–strain graph for precursor 32-layered fibers. (a_2_) 64-layered fibers. (a_3_) 128-layered fibers. (a_4_) 256-layered fibers. (Refer to Figure S2 for detailed stress–strain curve).

The concentration of GF in the composite significantly influences its mechanical properties, with optimal concentration leading to notable improvements in modulus and strength. For instance, in the 128-layered fibers (Figure 2a_3_), the modulus increased from 8.95 GPa (10PAN) to 16.93 GPa (10PAN-4GF), nearly doubling the stiffness due to the rigid GF acting as stiffening agents that restrict polymer chain mobility. Similarly, the modulus increased for both the 32 and 128-layered fibers as GF concentration increased, showing an overall improvement in mechanical properties with increased filler content across various layer configurations Figure 2. However, higher GF concentrations, such as 4 wt %, can sometimes introduce challenges, particularly in composites with 256 layers. Nonuniform dispersion at higher concentrations can create voids, agglomeration, or stress concentrators, which weaken the composite’s mechanical performance. For example, the stiffness of 10PAN-1GF (10.01 GPa) and 10PAN-4GF (10.26 GPa) fibers at 64 layers (Figure 2a_2_) exhibit only a minor variation, highlighting the importance of achieving uniform dispersion and avoiding defects. While increased GF content enhances stiffness and strength, it also reduces elongation at break, as seen in 32-layered fibers where elongation decreased from 12.28% to 8.64% with 4 wt % GF. This reduction in flexibility underscores the trade-off between rigidity and ductility, emphasizing the need for optimized GF concentration and effective manufacturing processes to balance reinforcement benefits and maintain structural integrity.

#### 2.2.3 The Fiber Layering Effects

The mechanical properties of layered composite fibers demonstrate that increasing the number of layers significantly enhances their performance by improving load distribution and stress management within the fiber matrix. For instance, the 256-layered composite fibers in Table 1 exhibit a remarkable increase in tensile strength and modulus compared to fibers with fewer layers. For 10PAN-1GF fibers, the modulus increases by 54.7%, from 15.10 GPa in pure PAN fibers to 23.37 GPa, while tensile strength improves by 27.2%, rising from 521.71 to 663.66 MPa. These improvements highlight the efficiency of solid waste derived from wind turbine blades as reinforcement fillers, with GF effectively enhancing the mechanical properties of multilayered composites. By spreading the load across a higher number of layers, the composites reduce stress concentrations and minimize the risk of failure under high loads. This resilience underscores the potential for utilizing wind turbine blade waste in more precisely controlled structural fibers to create advanced, high-performance composites.

However, increasing the number of layers also presents challenges, particularly in ensuring uniform GF dispersion and avoiding manufacturing defects. As seen in Figure 2a_4_, the 256-layered fibers with higher GF content (e.g., 10PAN-4GF) exhibit a decrease in tensile strength and modulus compared to their 10PAN-1GF counterparts. This suggests that nonuniform GF distribution and potential agglomeration in highly layered structures may introduce voids, cracks, or misalignments, which act as stress concentrators and weaken the composite’s mechanical performance. For example, the modulus of 10PAN-4GF fibers decreases to 14.24 GPa in 256-layered composites, compared to 23.37 GPa for 10PAN-1GF fibers. Despite these challenges, proper optimization of filler concentration and layer alignment can mitigate these defects (i.e., a balance between glass fiber content and layer thickness). By ensuring strong interfacial bonding between the matrix and the GF, along with precise manufacturing processes, the potential of high-layered composites to achieve superior mechanical properties is maximized, further validating the value of solid waste as an efficient reinforcement material.

### 2.3 SEM

The SEM images in Figure 3 reveal the fractured morphology of both as-spun and high-draw-ratio fibers with increasing layer numbers, highlighting the effects of layer structure and glass fiber reinforcement. As the layer number increases, the alternating layer width decreases, resulting in improved alignment of the GF along the fiber axis. In 32-layered fibers (Figure 3a_1_–a_4_), clear differences are observed between pure PAN and PAN-GF composite fibers. The as-spun pure PAN fibers exhibit distinct and uniform alternating layers (Figure 3a_1_), while the PAN-GF fibers show the integration of alternating PAN and PAN/GF layers, with color differentiation illustrating the presence of GF (Figure 3a_2_). The degree of GF alignment within the composite fibers is apparent in Figure 3a_3_, where SEM images demonstrate the qualitative orientation of the GF. However, the interference of GF within the PAN matrix leads to fiber rupture, followed by fiber pull-out, as seen in Figure 3a_4_. This pull-out mechanism creates voids and reduces mechanical performance, particularly in as-spun fibers.

**Figure 3:**
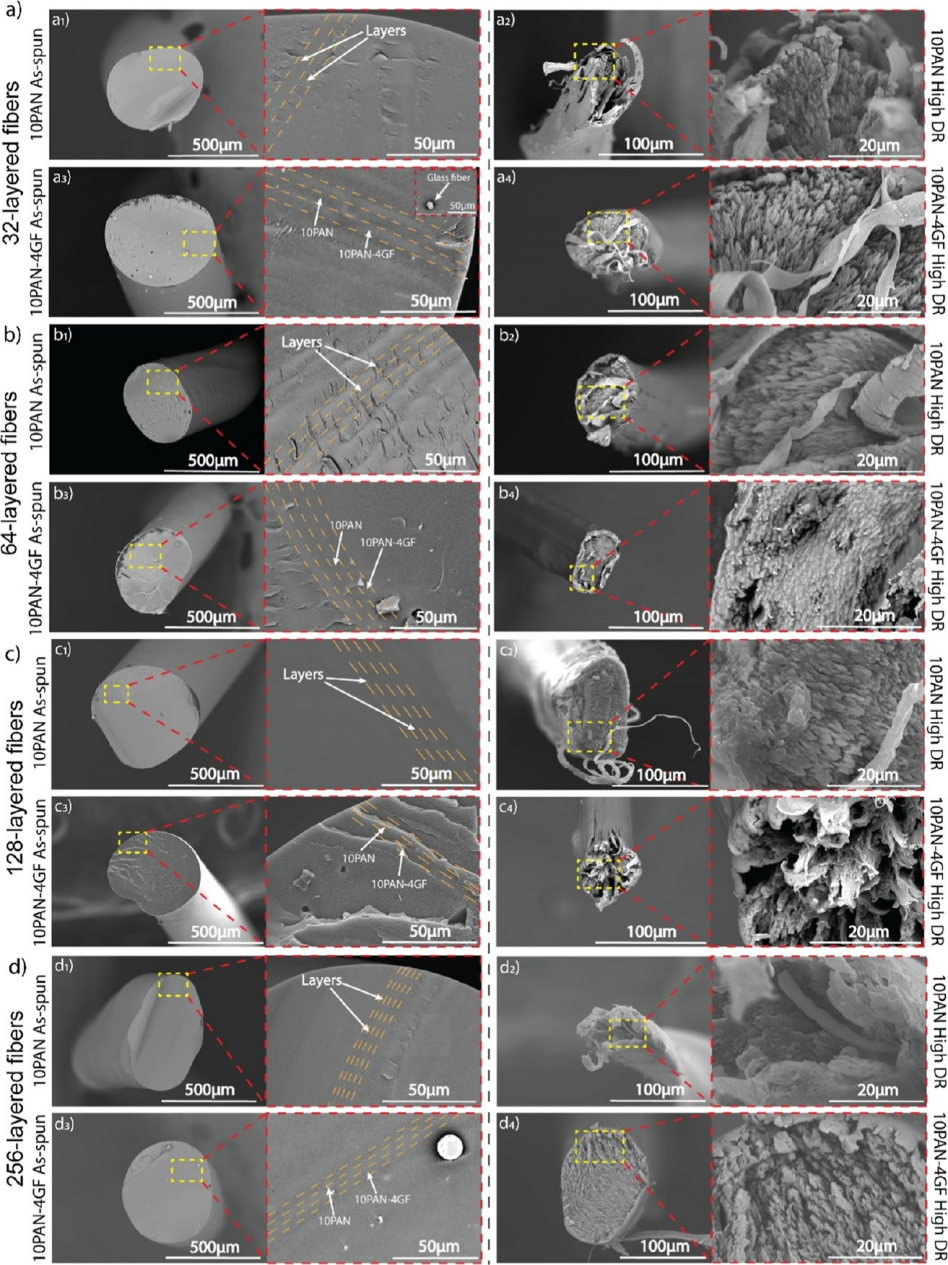
Fiber morphology of 10PAN and 10PAN-4GF fibers in their as-spun and high-draw-ratio (DR) states. (a) Cross-sectional and surface morphology of 32-layered fibers: (a_1_–a_4_) 10PAN and 10PAN-4GF. (b) Cross-sectional and surface morphology of 64-layered fibers: (b_1_–b_4_) 10PAN and 10PAN-4GF. (c) Cross-sectional and surface morphology of 128-layered fibers: (c_1_–c_4_) 10PAN and 10PAN-4GF. (d) Cross-sectional and surface morphology of 256-layered fibers: (d_1_–d_4_) 10PAN and 10PAN-4GF. Images reveal the layered structure and GF distribution along the fiber axis, with higher magnifications highlighting fiber alignment, internal defects, and surface characteristics. Scale bars: 500 μm, 100 μm, and 20 μm.

For 64-layered fibers (Figure 3b_1_–b_4_), the pull-out of GF during mechanical testing creates holes in the polymer matrix, which are evident in the as-spun fibers (Figure 3b_3_). These voids are larger and more irregular due to less organized fiber alignment in the as-spun state. However, high DR fibers (Figure 3b_4_) demonstrate enhanced alignment of GF and improved fiber–matrix interactions. This results in a more controlled pull-out process, with smaller and fewer voids in the matrix, contributing to superior mechanical properties and higher strength compared to as-spun fibers. The increased alignment of GF in the high DR fibers reduces stress concentration and enhances load transfer, improving their overall structural integrity.

In 128-layered fibers (Figure 3c_1_–c_4_), the higher concentration of GF in the composite (Figure 3c_4_) creates stress-concentration sites that make the fibers more prone to failure under mechanical stress. These agglomerates disrupt the uniform dispersion of GF within the matrix, leading to weaker interfacial bonding between the polymer matrix and the fillers. The resulting nonuniformity limits the anticipated improvements in mechanical performance, emphasizing the importance of achieving proper GF dispersion.

Finally, in 256-layered fibers (Figure 3d_1_–d_4_), the reduced thickness of alternating layers enhances GF alignment, as seen in Figure 3d_3_. This improved alignment results from the shear stress generated during fiber spinning, which promotes better orientation at the interfaces between layers. Under mechanical stress, fibrillar structures form in these fibers due to plastic deformation, as shown in Figure 3d_4_. The stretching and alignment of polymer chains along the fiber axis create elongated fibril-like structures that reinforce molecular alignment, contributing to the fiber’s strength and stiffness. The impact of GF concentration on these 256-layered fibers is further illustrated in Figure S3, showing the influence of GF wt % on the mechanical performance of the composites.

The structural features observed in Figure 3 strongly correlate with the mechanical properties presented in Table 1, highlighting the relationship between fiber morphology and performance metrics. As the number of layers increases, the alternating layer width decreases, resulting in enhanced GF alignment and better load distribution across the composite. For example, the 128-layered fibers demonstrate improved mechanical properties, with 10PAN-4GF fibers achieving a tensile strength of 449.17 MPa and a modulus of 16.93 GPa, compared to 350.20 MPa and 12.35 GPa, respectively, for pure 10PAN fibers. This improvement is attributed to the reduced thickness of alternating layers (Figure 3d_3_), which promotes greater shear stress during fiber spinning, enhancing GF alignment along the fiber axis. Additionally, fibrillar structures formed during mechanical stress in high-draw-ratio (DR) fibers (Figure 3d_4_) contribute to their strength and stiffness by reinforcing molecular alignment. However, imperfections such as voids and GF agglomerates, as observed in 128-layered fibers (Figure 3c_4_), disrupt uniform dispersion and weaken interfacial bonding, leading to a less significant modulus increase from 8.95 GPa (10PAN) to 16.93 GPa (10PAN-4GF). These structural features underscore the importance of optimizing layer numbers and GF dispersion to achieve maximum mechanical performance in composite fibers.

### 2.4 Thermogravimetric Analysis

TGA of pure PAN and PAN-GF composite fibers reveals that both the GF concentration and the number of layers significantly impact the thermal stability and residual weight of the fibers. These fibers undergo a significant weight loss between 300 and 400 °C, mainly due to cyclization and dehydrogenation reactions, as well as the release of hydrogen cyanide (HCN).^48^ The initial degradation temperatures range from ∼285 °C to ∼293 °C, and the final degradation (endset) temperatures reach up to ∼447 °C (Table S2). At lower concentrations of solid waste, such as in 10PAN-1GF fibers, the inconsistent control and distribution of GF can lead to variability in thermal performance. For example, in 32-layered fibers, the residual weight percentage for 10PAN-1GF (34.96 wt %) is lower than for 10PAN-4GF (35.20 wt %), indicating a lack of uniform GF distribution when the GF concentration is minimal, as seen in Figure 4a_1_. This variability can undermine the reinforcement effect of GF, as nonuniform dispersion creates localized weak points that may compromise the fiber’s thermal and mechanical properties.

**Figure 4:**
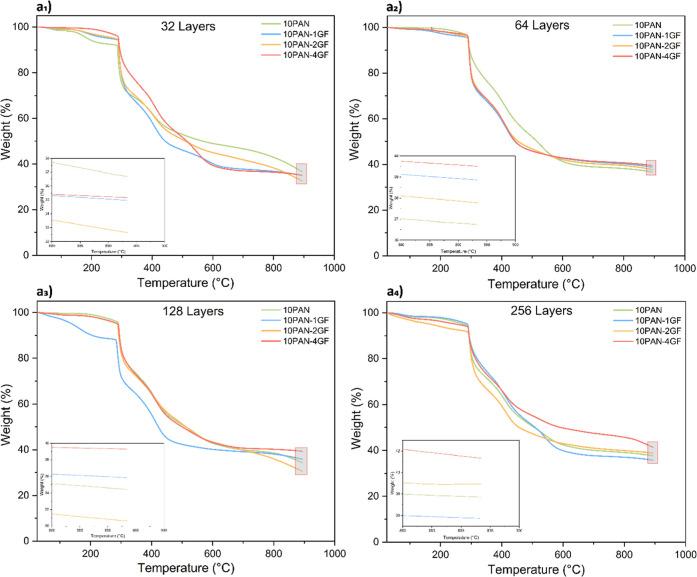
Thermogravimetric analysis for the precursor fibers of pure PAN and composites containing varying glass fiber concentrations. (a_1_) 32-layered fibers (a_2_) 64-layered fibers (a_3_) 128-layered fibers (a_4_) 256-layered fibers.

In contrast, when the GF concentration and layer number increase, as seen in 256-layered fibers, the uniform distribution of GF contributes to more consistent thermal performance and improved loading reliability in experimental designs. For example, the residual weight percentage increases progressively with GF concentration, from 36.66 wt % in pure 10PAN fibers to 41.33 wt % in 10PAN-4GF fibers. This trend reflects the reinforcing effect of uniformly distributed GF, which enhances the composite’s thermal stability by resisting degradation processes. Moreover, higher-layered composites with higher GF concentrations demonstrate multiple decomposition stages, as evident in Figure 4a_4_, showcasing their ability to maintain structural integrity at elevated temperatures. Thus, increasing the GF concentration and optimizing their uniform distribution in high-layered composites enhances both their thermal properties and the reproducibility of their mechanical performance.

### 2.5 Heat Treatment of the Fibers

During the heat-treatment process of PAN-based fibers, temperature plays a crucial role in determining the quality of carbonized fibers. Stabilization, typically occurring between 200 and 300 °C, is critical to the transformation of PAN into a thermally stable structure.^31^ Excessive stabilization temperatures, as indicated in the literature, can lead to overheating, fusion, or even combustion of fibers, compromising their mechanical and structural properties. On the other hand, insufficient stabilization temperatures result in incomplete reactions, hindering proper stabilization and negatively affecting carbon fiber quality.^49,50^ Optimal stabilization temperature, such as 260 °C (optimized in our experiments), can minimize these risks by facilitating dehydrogenation and cross-linking, which is essential for carbon fiber formation (Figure S4).^51^ Shrinkage during stabilization, while undesirable, is often minimized by stretching fibers at a constant length to maintain the linear alignment of polymer chains, which is vital for achieving high-performance mechanical properties.^52^ Nevertheless, some shrinkage can still occur due to fiber slippage, which can affect the fiber morphology and contribute to the formation of voids and defects (Figure S5).

Carbonization further transforms the stabilized PAN matrix into a carbon-rich structure (Figure S4).^53,54^ The WTB solid waste within the composite fiber begins to degrade at approximately 500 °C–700 °C (resins).^55^ As a result, the carbonization temperature must be set lower (700 °C optimized in our experiments) to prevent premature decomposition and ensure material integrity during the heat treatment process (Figure S5). However, excessive temperatures can also induce localized volume changes, forming voids or defects that degrade composite properties. During carbonization, the GF within the composite fibers enhances the alignment of PAN chains. This enhanced alignment improves crystallinity and facilitates dehydrogenation, resulting in high-performance CF.

SEM analysis of carbonized fibers (Figures 5a and S6) reveals distinct morphological features between 256 layered CF of pure PAN and PAN-GF fibers. Pure PAN fibers exhibit voids and cracks on the cross-section (Figure 5a_1_,a_2_), reflecting crack propagation during heat treatment. Elemental analysis via EDS (Figure 5b_1_–c_1_) confirms that these fibers are composed primarily of carbon (approximately 79 wt %) with faint traces of oxygen due to surface oxidation. In contrast, PAN-GF carbonized fibers maintain an intact interphase between the GF and PAN matrix after carbonization (Figure 5a_3_,a_4_). EDS mapping (Figure 5b_2_) and spectra (Figure 5c_2_) further confirm the presence of silica (17 wt %) and calcium (3.5 wt %) in the composite, along with carbon (70 wt %). The uniform dispersion of GF contributes to the thermal and structural stability of the composite, reinforcing the fiber’s mechanical properties and making it suitable for high-performance applications.

**Figure 5:**
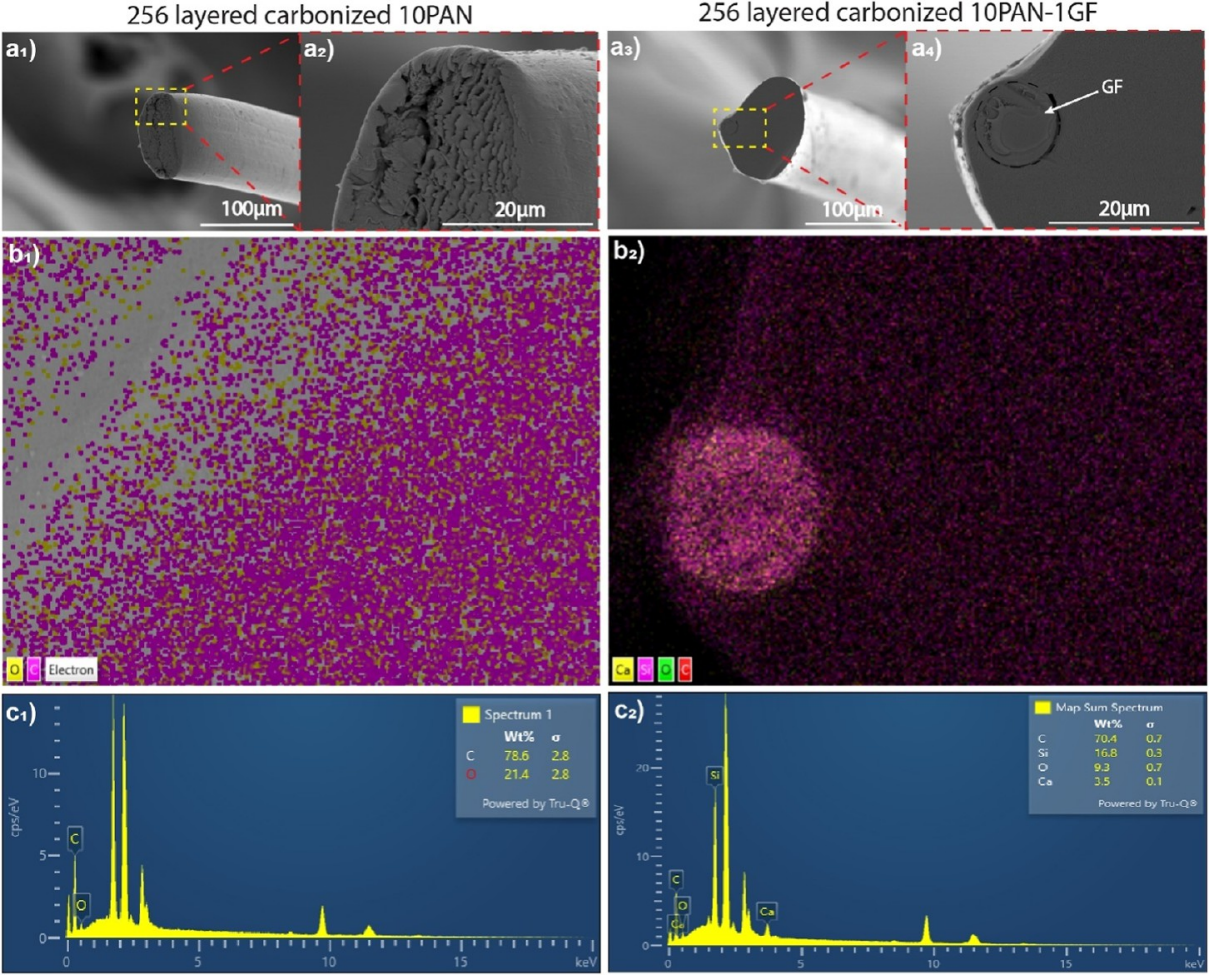
SEM morphology and EDS results of carbonized fibers. SEM image of (a_1_,a_2_) PAN carbonized fiber and (a_3_,a_4_) PAN-GF carbonized fiber. EDS mapping (b_1_,b_2_). EDS quantitative composition for carbonized fibers of PAN (c_1_) and PAN-GF (c_2_).

### 2.6 Carbonized Fiber Applications

#### 2.6.1 Thermal Properties of Carbonized Fibers

Thermogravimetric analysis of CF was conducted to evaluate their thermal stability and residual mass retention at high temperatures. The TGA results (Figure S7) indicate that the CF retained 83 wt % of their mass at 900 °C, demonstrating excellent resistance to thermal decomposition. This high residual weight highlights the minimal degradation of CF even under extreme thermal conditions, making them suitable for high-temperature applications. The inclusion of GF further enhances thermal stability, as evidenced by the consistent mass retention trends across the analyzed samples.

The vertical burn test of CF, performed according to ASTM D6413 standards, further confirmed the exceptional flame resistance of the material (Figure 6a). Under continuous flame exposure of approximately 1900 °C (propane gas) for 40 s, the CF demonstrated minimal shrinkage or deformation, maintaining structural integrity throughout the test. This behavior underscores the material’s superior thermal stability and flame resistance, which are critical for applications in extreme environments, such as aerospace and defense. In contrast, precursor fibers exhibit significantly lower thermal stability, as shown in the comparative analysis (Figure S8), where they degrade rapidly under similar conditions. These findings highlight the advanced thermal performance of CF, making them an ideal candidate for high-performance applications requiring durability at elevated temperatures.

**Figure 6:**
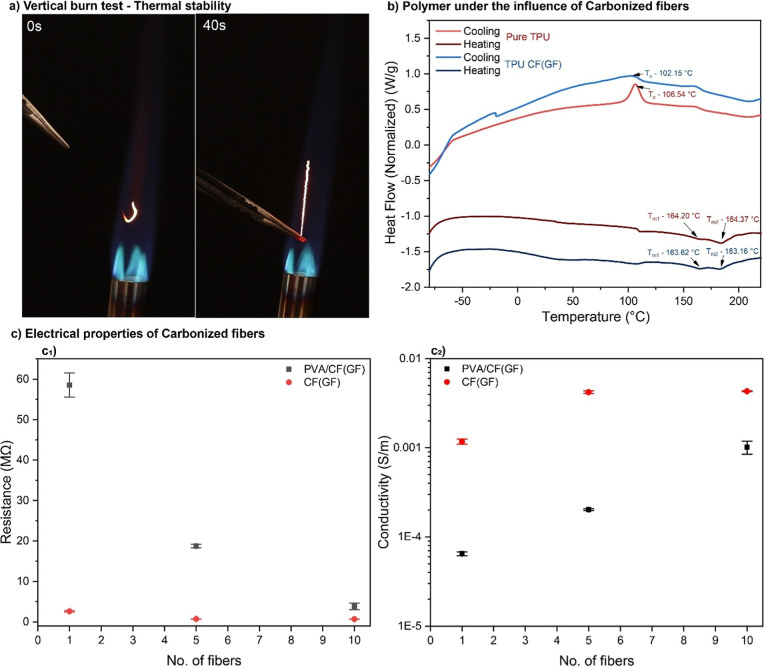
Applications of the carbonized fibers with the (a) vertical burn test (ASTM D6413) to determine the thermal stability, (b) polymer behavior under the influence of carbonized fibers, (c) electrical properties of the carbonized fibers and polymer composites, (c_1_) resistance with increasing fiber numbers, and (c_2_) conductivity along the fiber axis with increasing fiber numbers.

#### 2.6.2 Polymer under the Influence of Carbonized Fibers

Carbon fibers can be templates for polymer crystallization that may benefit the interfacial interactions in polymer composites. The differential scanning calorimetry analysis of pure TPU and TPU/CF­(GF) (Figure 6b) highlights the significant influence of carbonized fibers on the thermal behavior and crystallinity of the polymer. The crystallization temperature (*T*_c_) decreases from 106.54 °C for pure TPU to 102.15 °C for TPU/CF­(GF), indicating that CF acts as a nucleating agent, facilitating the early formation of crystalline domains. This reduction in *T*_c_ reflects enhanced crystallization kinetics, as the CF provides nucleation sites that promote faster and more controlled crystalline growth. Additionally, the melting temperatures (*T*_m1_ and *T*_m2_) of TPU/CF­(GF) also exhibit a slight shift compared to those of pure TPU. The first melting temperature (*T*_m1_) decreases from 164.20 °C in pure TPU to 163.62 °C in TPU/CF­(GF), while the second melting temperature (*T*_m2_) decreases slightly from 184.37 to 183.16 °C. This behavior suggests that CF influences the thermal transitions by modifying the crystal structure and distribution within the polymer matrix. The improved crystallization observed in TPU/CF­(GF) compared to pure TPU (Figure S9) underscores the role of CF in enhancing polymer crystallinity, which can lead to improved mechanical and thermal properties in composite systems.

#### 2.6.3 Electrical Conductivity of Fibers

The electrical properties of CF­(GF) and PVA/CF­(GF) composites were evaluated along the fiber axis, as shown in Figure S10. The trends in electrical resistivity and conductivity with increasing fiber numbers are depicted in Figure 6c. CF­(GF) fibers exhibited superior electrical conductivity compared to PVA/CF­(GF) composites due to the electron scattering at the presence of an insulating polymer matrix, which limits current flow. Also, the resistivity of PVA/CF­(GF) fibers decreased from 1.54 × 10^–2^ Ω m for a single fiber to 1.02 × 10^–2^ Ω m for a 10-fiber bundle (Table S3). This reduction results from enhanced parallel conductive paths, minimized contact resistance, and improved alignment of fibers in the bundle. Correspondingly, the conductivity increased from 6.47 × 10^–5^ S/m for a single fiber to 1.01 × 10^–3^ S/m for a 10-fiber bundle. In contrast, CF­(GF) fibers showed significantly lower resistivity, decreasing from 8.55 × 10^–4^ Ω m for a single fiber to 2.31 × 10^–4^ Ω m for a 10-fiber bundle, with corresponding conductivity values of 1.17 × 10^–3^ S/m and 4.32 × 10^–3^ S/m, respectively. While bundling initially introduces minor resistance due to imperfect contact (Figure S11), the addition of more fibers provides additional conductive pathways, optimizing overall electrical performance in larger bundles.^56^ These results highlight the potential of CF­(GF) fibers for applications requiring high electrical conductivity.

## 3 Conclusion

This study underscores the potential of repurposing solid waste from mechanically recycled wind turbine blades as a filler material in PAN-GF composites, addressing both environmental sustainability and advancing high-performance composite fibers. The finer-diameter fibers achieved in this work demonstrate enhanced composite material performance by reducing defects and ensuring proper alignment of glass fibers within the layers, resulting in significantly improved mechanical properties. As the number of layers increased in the multilayered PAN-GF fibers, the thickness of each layer progressively decreased, with 32-layered fibers having relatively thicker layers and 256-layered fibers exhibiting much thinner ones. TGA analysis shows that the residual weight at 900 °C rises with higher GF content, demonstrating improved thermal stability and char formation. Notably, the 10PAN-4GF fiber with 256 layers exhibited the highest residual mass (41.23 wt %), underscoring the role of GF reinforcement in enhancing thermal stabilization. The 256-layered 10PAN-1GF composite fibers, the stiffness (modulus) increased by 55% (i.e., from 15.10 to 23.37 GPa), while the tensile strength improved by 27% (i.e., from 521.71 to 663.66 MPa). These improvements were facilitated by the uniform layering of PAN and PAN-GF, ensuring robust structural integrity. Additionally, the precursor fibers were heat-treated in an inert atmosphere to produce carbonized fibers, which exhibit exceptional thermal stability, enabling them to withstand high temperatures. Carbonized fibers also enhance the polymer’s crystallization behavior, promoting faster and more efficient crystallization, further broadening their applicability in advanced composite systems.

## 4 Experimental Section

### 4.1 Materials

The PAN copolymer used in this study consisted of 99.5% acrylonitrile and 0.5% methacrylate, with a molecular weight of 230,000 g/mol and an average particle size of 50 μm, sourced from Goodfellow Cambridge Limited, Huntingdon, England. The solid waste from the WTB was obtained from TPI Composites, Inc. Iowa, USA. These WTBs consisting of wood, adhesives, coatings, and GF, were processed by breaking down the composite material and reducing particle size through processes such as shredding, crushing, milling, grinding, and sieving (through mesh 40) to produce fine particles with 82 wt % GF concentration (Figure S12). ImageJ software was used to estimate the average particle size of processed GFRP, which was determined to be 38 μm in length and 4 μm in diameter. The solvents used included *N*,*N*-dimethylformamide (DMF) (ACS reagent, ≥99.8%) to dissolve PAN and disperse GF, and methanol (ACS reagent, ≥99.8%) as a coagulant, both obtained from Sigma-Aldrich, USA. All materials were purchased and used as received, without further modifications.

### 4.2 Multilayered Fiber Spinning

The following section describes the spinning of multilayered fibers.

Preparation of multilayered PAN and PAN-GF feedstocks: Preparation of fiber-spinner feedstock. Eight g of PAN were dissolved in 80 mL of DMF to create the feedstock. Initially, GF was first incorporated at concentrations of 1–4 wt % w.r.t PAN in the DMF solvent, resulting in a suspension achieved by tip sonication for 30 min at an amplitude of 60% (Q500, Fisher Scientific, US). Subsequently, PAN was added to the solvent to create the PAN-GF composite solution for feedstock B. Both mixtures were stirred mechanically at 130 °C for 45 min until a clear solution was achieved. To eliminate air bubbles, the solutions were placed in a vacuum oven (Lindberg Blue M lab oven, Thermo Scientific US) at 50 °C for 30 min. Following deaeration, the solutions were transferred to a metal syringe connected to a pump for fiber spinning, which were injected into a multilayered spinneret at a controlled rate of 2 mL/min to facilitate the extrusion and formation of fibers. This unique multilayered spinneret was manufactured using a Concept Laser 2 metal 3D printer using Inconel, allowing for intricate designs and precise control in the fiber extrusion process.

Dry-jet wet spinning of multilayered PAN and PAN-GF fibers:

#### 4.2.1 Fiber Spinning

The solution was injected into an air gap of 1.5–2.0 cm before entering the coagulation bath. In the dry-jet wet spinning, the air gap facilitated fiber extension, reducing defect density and promoting molecular alignment.^57^ Immersion in the coagulation bath triggered two simultaneous diffusion processes: the polymer-rich phase condensed into the fiber, while the solvent-rich phase (DMF) exchanged with the nonsolvent (methanol), forming a gel-like fiber structure. The as-spun fibers were then soaked in methanol for 30 min for coagulation. The coagulation rate needed to be high enough to minimize gradient differences between the surface and core, ensuring a uniform coagulation procedure and preventing core deformation, resulting in a circular fiber shape. However, irregular cross-sectional shapes could develop if diffusion rates between layers are mismatched, causing a gradient in the polymer distribution. Flow and injection rates were critical in determining fiber dimensions and chain alignment.^58,59^ Higher flow rates through the coagulation bath and lower injection rates for the spinning solution resulted in a lower fiber diameter and higher defect density. However, a larger draw ratio during coagulation did not always guarantee better polymer chain alignment because of stretching and recoiling effects.^60^ For example, excessive stretching from high flow rates could lead to molecular recoil, hindering further polymer chain alignment.^61^ Therefore, the injection rates were optimized at 2 mL/min for collection onto the winders. Table 2 summarizes the fibers with varying layer numbers, consisting of alternating PAN-GF layers, with layer numbers ranging from 32 to 256 layers. It also shows the impact of incorporating GF and its fiber stretchability and dimensions with pure PAN fibers, highlighting the effects of increased GF content on the structural properties that are consistent for fibers with varying DR.

**Table 2:** Table Summarizes the Layer Numbers, Their Compositions, and Drawing Capabilities

		composition wt %		drawing results		
layers	fiber type	feedstock A (PAN wt %)	feedstock B (GF wt % w.r.t PAN)	processing of fibers (drawing and heat-treatment)	diameter of manually stretched fiber (μm)	individual layer width (μm)
32	10PAN	10	0	precursor fibers were drawn in water at 85 °C and in silicone oil at 125 °C, 135 °C, and 145 °C. The drawn fibers were subsequently carbonized in a tube furnace at 700 °C to produce carbonized fibers	58	1.81
	10PAN-1GF		1		60	1.87
	10PAN-2GF		2		61	1.90
	10PAN-4GF		4		58	1.81
64	10PAN		0		60	0.93
	10PAN-1GF		1		55	0.86
	10PAN-2GF		2		61	0.95
	10PAN-4GF		4		50	0.78
128	10PAN		0		51	0.40
	10PAN-1GF		1		51	0.40
	10PAN-2GF		2		55	0.43
	10PAN-4GF		4		57	0.44
256	10PAN		0		51	0.19
	10PAN-1GF		1		52	0.20
	10PAN-2GF		2		51	0.19
	10PAN-4GF		4		53	0.20

#### 4.2.2 Fiber Drawing

During hot drawing, fibers were stretched through baths of water and silicone oil to their maximum draw ratios without breaking. High shear forces aligned the macromolecules parallel to the fiber axis.^62^ Initially, the fibers were drawn through a water bath at 85 °C to facilitate polymer chain alignment and remove DMF solvent. They were then soaked in methanol for 24 h to enhance the coagulation and minimize the DMF content in gel fibers. The wet PAN fibers were dried at 50 °C under vacuum for 30 min to remove moisture and collapse voids. Next, the fibers were drawn in an oil bath at 145 °C to produce the highest draw ratio fibers. This high-temperature drawing maximized molecular extension and applied a protective layer to the fibers. The increasing bath temperatures helped orient the GF by overcoming the rotational momentum of the GF.^63^ Stretching at higher temperatures also enhanced PAN fiber molecular orientation and created dense structures with improved mechanical properties.^64^

#### 4.2.3 Fiber Annealing

Fibers were heat-treated in a tube furnace (Lindberg Blue M, Thermo Scientific, US) in an oxidative atmosphere, heating at a rate of 1 °C/min to 260 °C, where they were held for 45 min to produce stabilized fibers. Subsequently, these stabilized fibers were heated at a rate of 1 °C/min in a nitrogen atmosphere with a gauge pressure of 0.01 kPa to 700 °C, with a dwell time of 30 min, to produce CF. Finally, the fibers were allowed to be cooled to room temperature at a rate of 5 °C/min.

### 4.3 Characterizations

Single fibers were subjected to tensile testing using a tensile tester (Discovery HR-2 hybrid rheometer, TA Instruments Inc., USA) with a gauge length of 20 mm and a constant linear strain rate of 50 μm/s for the highest drawn fibers. 8–10 samples of each fiber type were tested to obtain mechanical parameters, including Young’s modulus, tensile strength, and tensile strain.

The fiber morphology of various PAN and PAN-GF layered fibers, with different GF concentrations, was examined using Thermo Fisher Scientific (FEI) Teneo, a field emission scanning electron microscope (FESEM) at an operating voltage of 15 kV. All fibers were mounted on a 90° cross-sectional stub with the fractured surface facing up and coated with a thin gold layer (30 nm) from the Leica EM ACE600 Coater.

Thermogravimetric analysis (TGA) (TGA 550, TA Instruments Inc., USA) was conducted on 2 mg fiber samples from each fiber type, with the temperature raised to 900 °C at a heating rate of 10 °C/min in a nitrogen atmosphere, to analyze thermal stability and decomposition behavior.

Vertical burn test method (ASTM D6413), a Bunsen burner flame was applied to the fibers for 40 s for the sample, using a 25 mm sample to assess the thermal stability of the fiber.

Differential scanning calorimetry (DSC) analyses were conducted using a DSC 250 (TA Instruments Inc., USA) on samples weighing between 0.30 and 0.50 mg. The measurements were performed over a temperature range of −90 to 240 °C under a nitrogen atmosphere to investigate the melting and crystallization behavior of both pure polymers and carbon fiber (CF) composites.

The electrical conductivity of the CF­(GF) (each fiber length-15.5 mm) and polymer–CF­(GF) (each fiber length-19 mm) with approximately 80 μm diameters was measured along the fiber axis using a two-probe multimeter. Copper wires were attached to the probes to ensure uniform surface contact, and a conductive silver paste was applied to establish a connection between the sample and the wires linked to the instrument. The resistivity (eq 1) and conductivity (eq 2) of the fibers were obtained using the following equations.

1 $$\rho =R\times \frac{A}{L}$$

2 $$\sigma =\frac{1}{\rho }$$

where ρ is resistivity (Ω m), *R* is resistance (Ω), *A* is the area of the fiber (m^2^), *L* is the fiber sample length (m), and σ is conductivity (S/m).

## Supplementary Material





## Notes

Additional information includes fiber spinning conditions and drawability; mechanical analysis of precursor fibers; cross-sectional morphologies of 256-layered precursor fiber; thermogravimetric analysis (TGA) of precursor composite fibers to assess degradation temperatures and residual weight; heat treatment processes including stabilization in air and carbonization in nitrogen to produce carbonized fibers; cross-sectional morphologies of 256-layered carbonized fibers; TGA of carbonized fibers and polymer–carbon fiber composites; thermal stability of precursor fibers via vertical burn test (ASTM D6413); polymer behavior influenced by embedded carbonized fibers; and electrical properties of the resulting carbonized fibers; TGA of mechanically recycled wind turbine blades to determine glass fiber concentration (PDF)
Vertical burn test (ZIP)

## References

[1] Geyer R., Jambeck J. R., Law K. L. (2017). Production,
Use, and Fate of All
Plastics Ever Made. Sci. Adv..

[2] Ellis L. D., Rorrer N. A., Sullivan K. P., Otto M., McGeehan J. E., Román-Leshkov Y., Wierckx N., Beckham G. T. (2021). Chemical and Biological
Catalysis for Plastics Recycling and Upcycling. Nat. Catal..

[3] Rosenboom J. G., Langer R., Traverso G. (2022). Bioplastics
for a Circular Economy. Nat. Rev. Mater..

[4] Yudell M., Roberts D., DeSalle R., Tishkoff S. (2020). NIH Must Confront the
Use of Race in Science. Science.

[5] Anshassi M., Townsend T. G. (2023). The Hidden Economic
and Environmental Costs of Eliminating
Kerb-Side Recycling. Nat. Sustain..

[6] Ballout W., Sallem-Idrissi N., Sclavons M., Doneux C., Bailly C., Pardoen T., Van Velthem P. (2022). High Performance Recycled CFRP Composites
Based on Reused Carbon Fabrics through Sustainable Mild Solvolysis
Route. Sci. Rep..

[7] Pan S. Y., Chen Y. H., Fan L. S., Kim H., Gao X., Ling T. C., Chiang P. C., Pei S. L., Gu G. (2020). CO2 mineralization
and Utilization by Alkaline Solid Wastes for Potential Carbon Reduction. Nat. Sustain..

[8] Sharma S., Kalita G., Hirano R., Shinde S. M., Papon R., Ohtani H., Tanemura M. (2014). Synthesis of Graphene
Crystals from
Solid Waste Plastic by Chemical Vapor Deposition. Carbon.

[9] Wang C., Li D., Zhai T., Wang H., Sun Q., Li H. (2019). Direct Conversion
of Waste Tires into Three-Dimensional Graphene. Energy Storage Mater..

[10] Rani M., Choudhary P., Krishnan V., Zafar S. (2021). A Review on Recycling
and Reuse Methods for Carbon Fiber/Glass Fiber Composites Waste from
Wind Turbine Blades. Composites, Part B.

[11] Beauson J., Laurent A., Rudolph D. P., Pagh Jensen J. (2022). The Complex
End-of-Life of Wind Turbine Blades: A Review of the European Context. Renew. Sustain. Energy Rev..

[12] Cherrington R., Goodship V., Meredith J., Wood B. M., Coles S. R., Vuillaume A., Feito-Boirac A., Spee F., Kirwan K. (2012). Producer Responsibility:
Defining the Incentive for Recycling Composite Wind Turbine Blades
in Europe. Energy Policy.

[13] Fonte R., Xydis G. (2021). Wind Turbine Blade Recycling: An
Evaluation of the European Market
Potential for Recycled Composite Materials. J. Environ. Manage..

[14] Musial, W. ; Spitsen, P. ; Duffy, P. ; Beiter, P. ; Shields, M. ; Mulas Hernando, D. ; Hammond, R. ; Marquis, M. ; King, J. ; Sathish, S. Offshore Wind Market Report: 2023, 2023.

[15] Shields, M. ; Stefek, J. ; Oteri, F. ; Kreider, M. ; Gill, E. ; Maniak, S. ; Gould, R. ; Malvik, C. ; Tirone, S. ; Hines, E. A Supply Chain Road Map for Offshore Wind Energy in the United States, 2023.

[16] Qureshi J. (2022). A Review of
Recycling Methods for Fibre Reinforced Polymer Composites. Sustainability.

[17] Utekar S., V K S., More N., Rao A. (2021). Comprehensive Study of Recycling
of Thermosetting Polymer Composites - Driving Force, Challenges and
Methods. Composites, Part B.

[18] Shen M. Y., Guo Z. H., Feng W. T. (2023). A Study on the Characteristics and
Thermal Properties of Modified Regenerated Carbon Fiber Reinforced
Thermoplastic Composite Recycled from Waste Wind Turbine Blade Spar. Composites, Part B.

[19] Wei Y., Hadigheh S. A. (2022). Cost Benefit and
Life Cycle Analysis of CFRP and GFRP
Waste Treatment Methods. Constr. Build. Mater..

[20] Sobczak M. T., Li G., Ramanathan A., Thummalapalli S. V., Thippanna V., Chambers L. B., Theobald T., Sun H., Nolet S., Li K., Song K. (2025). Life Cycle Analysis
of Coaxial Layered Fiber Spinning
for Wind Turbine Blade Recycling. ACS Sustainable
Resour. Manage..

[21] Khalid M. Y., Arif Z. U., Hossain M., Umer R. (2023). Recycling
of Wind Turbine
Blades through Modern Recycling Technologies: A Road to Zero Waste. Renew. Energy Focus.

[22] Lin J., Guo Z., Hong B., Xu J., Fan Z., Lu G., Wang D., Oeser M. (2022). Using Recycled
Waste Glass Fiber
Reinforced Polymer (GFRP) as Filler to Improve the Performance of
Asphalt Mastics. J. Clean. Prod..

[23] Zhang Y., Pontikes Y., Lessard L., Willem van
Vuure A. (2021). Recycling
and Valorization of Glass Fibre Thermoset Composite Waste by Cold
Incorporation into a Sustainable Inorganic Polymer Matrix. Composites, Part B.

[24] Choi D., Kil H. S., Lee S. (2019). Fabrication
of Low-Cost Carbon Fibers
Using Economical Precursors and Advanced Processing Technologies. Carbon.

[25] Xu Z., Gao C. (2015). Graphene Fiber: A New
Trend in Carbon Fibers. Mater. Today.

[26] Dinu R., Lafont U., Damiano O., Orange F., Mija A. (2023). Recyclable,
Repairable, and Fire-Resistant High-Performance Carbon Fiber Biobased
Epoxy. ACS Appl. Polym. Mater..

[27] Awad R., Haghighat Mamaghani A., Boluk Y., Hashisho Z. (2021). Synthesis and Characterization
of Electrospun PAN-Based Activated Carbon Nanofibers Reinforced with
Cellulose Nanocrystals for Adsorption of VOCs. Chem. Eng. J..

[28] Pradere C., Batsale J. C., Goyhénèche J. M., Pailler R., Dilhaire S. (2009). Thermal Properties of Carbon Fibers
at Very High Temperature. Carbon.

[29] Zhang Y., Tajaddod N., Song K., Minus M. L. (2015). Low Temperature
Graphitization of Interphase Polyacrylonitrile (PAN). Carbon.

[30] Gao Z., Zhu J., Rajabpour S., Joshi K., Kowalik M., Croom B., Schwab Y., Zhang L., Bumgardner C., Brown K. R., Burden D., Klett J. W., van Duin A. C. T., Zhigilei L. V., Li X. (2020). Graphene Reinforced
Carbon Fibers. Sci. Adv..

[31] Rahaman M. S. A., Ismail A. F., Mustafa A. (2007). A Review of Heat Treatment
on Polyacrylonitrile
Fiber. Polym. Degrad. Stab..

[32] Rajabpour S., Mao Q., Gao Z., Khajeh Talkhoncheh M., Zhu J., Schwab Y., Kowalik M., Li X., van Duin A. C. T. (2021). Low-Temperature
Carbonization of Polyacrylonitrile/Graphene Carbon Fibers: A Combined
ReaxFF Molecular Dynamics and Experimental Study. Carbon.

[33] Liu Y., Yu X., Guo Y., Ren Y., Liu X. (2023). Preparation of Flame
Retardant, Smoke Suppression and Reinforced Polyacrylonitrile Composite
Fiber by Using Fully Biomass Intumescent Flame Retardant System and
Its Sustainable Recycle Application. Compos
Part A Appl. Sci. Manuf..

[34] Ravichandran D., Dmochowska A., Sundaravadivelan B., Thippanna V., Motta de Castro E., Patil D., Ramanathan A., Zhu Y., Sobczak M. T., Asadi A., Peixinho J., Miquelard-Garnier G., Song K. (2024). 3D Printing Carbon-Carbon Composites with Multilayered Architecture
for Enhanced Multifunctional Properties. J.
Mater. Chem. A.

[35] Song K., Zhang Y., Minus M. L. (2015). Polymer Interphase Self-Reinforcement
and Strengthening Mechanisms in Low-Loaded Nanocomposite Fibers. Macromol. Chem. Phys..

[36] Song K., Zhang Y., Minus M. L. (2014). Using Low Concentrations of Nano-Carbons
to Induce Polymer Self-Reinforcement of Composites for High-Performance
Applications. Mater. Res. Soc. Symp. Proc..

[37] Ramanathan A., Thippanna V., Kumar A. S., Sundaravadivelan B., Zhu Y., Ravichandran D., Yang S., Song K. (2024). Highly Loaded Carbon
Fiber Filaments for 3D-Printed Composites. J.
Polym. Sci..

[38] Hameed N., Sharp J., Nunna S., Creighton C., Magniez K., Jyotishkumar P., Salim N. V., Fox B. (2016). Structural
Transformation of Polyacrylonitrile Fibers during Stabilization and
Low Temperature Carbonization. Polym. Degrad.
Stab..

[39] Zhang Y., Song K., Meng J., Minus M. L. (2013). Tailoring Polyacrylonitrile
Interfacial Morphological Structure by Crystallization in the Presence
of Single-Wall Carbon Nanotubes. ACS Appl. Mater.
Interfaces.

[40] Ravichandran D., Xu W., Kakarla M., Jambhulkar S., Zhu Y., Song K. (2021). Multiphase
Direct Ink Writing (MDIW) for Multilayered Polymer/Nanoparticle Composites. Addit. Manuf.

[41] Jambhulkar S., Ravichandran D., Thippanna V., Patil D., Song K. (2023). A Multimaterial
3D Printing-Assisted Micropatterning for Heat Dissipation Applications. Adv. Compos Hybrid Mater..

[42] Xu W., Jambhulkar S., Ravichandran D., Zhu Y., Lanke S., Bawareth M., Song K. (2022). A Mini-Review of Microstructural
Control during Composite Fiber Spinning. Polym.
Int..

[43] Xu W., Franklin R., Ravichandran D., Bawareth M., Jambhulkar S., Zhu Y., Kakarla M., Ejaz F., Kwon B., Hassan M. K., Al-Ejji M., Asadi A., Chawla N., Song K. (2022). Continuous
Nanoparticle Patterning Strategy in Layer-Structured Nanocomposite
Fibers. Adv. Funct. Mater..

[44] Jambhulkar S., Ravichandran D., Zhu Y., Thippanna V., Ramanathan A., Patil D., Fonseca N., Thummalapalli S. V., Sundaravadivelan B., Sun A., Xu W., Yang S., Kannan A. M., Golan Y., Lancaster J., Chen L., Joyee E. B., Song K. (2024). Nanoparticle Assembly:
From Self-Organization to Controlled Micropatterning for Enhanced
Functionalities. Small.

[45] Chae H. G., Kumar S. (2008). Materials Science: Making Strong Fibers. Science.

[46] Xu W., Ravichandran D., Jambhulkar S., Zhu Y., Song K. (2021). Hierarchically
Structured Composite Fibers for Real Nanoscale Manipulation of Carbon
Nanotubes. Adv. Funct. Mater..

[47] Griffits A. A. V. I. (1921). The
Phenomena of Rupture and Flow in Solids. Philos.
Trans. R. Soc., A.

[48] Sun X., Li X., Thippanna V., Doyle C., Mu Y., Barrett T., Chambers L. B., Yu C., Levendis Y., Song K., Minus M. (2025). Carbon Nanoparticle
Effects on PAN Crystallization for Higher-Performance
Composite Fibers. ACS Polym. Au.

[49] Minus M. L., Kumar S. (2005). The Processing, Properties,
and Structure of Carbon Fibers. JOM.

[50] Kumar S., Doshi H., Srinivasarao M., Park J. O., Schiraldi D. A. (2002). Fibers
from Polypropylene/Nano Carbon Fiber Composites. Polymer.

[51] Konstantopoulos G., Soulis S., Dragatogiannis D., Charitidis C. (2020). Introduction
of a Methodology to Enhance the Stabilization Process of PAN Fibers
by Modeling and Advanced Characterization. Materials.

[52] Chae H. G., Minus M. L., Rasheed A., Kumar S. (2007). Stabilization and Carbonization
of Gel Spun Polyacrylonitrile/Single Wall Carbon Nanotube Composite
Fibers. Polymer.

[53] Pramanik C., Jamil T., Gissinger J. R., Guittet D., Arias-Monje P. J., Kumar S., Heinz H. (2019). Polyacrylonitrile
Interactions with
Carbon Nanotubes in Solution: Conformations and Binding as a Function
of Solvent, Temperature, and Concentration. Adv. Funct. Mater..

[54] Lu M., Arias-Monje P. J., Ramachandran J., Gulgunje P. V., Luo J., Kirmani M. H., Meredith C., Kumar S. (2021). Stabilization of Polyacrylonitrile
Fibers with Carbon Nanotubes. Polym. Degrad.
Stab..

[55] Xu M. x., Ji H. w., Wu Y. c., Di J. y., Meng X. x., Jiang H., Lu Q. (2023). The Pyrolysis
of End-of-Life Wind
Turbine Blades under Different Atmospheres and Their Effects on the
Recovered Glass Fibers. Composites, Part B.

[56] Thummalapalli S. V., Patil D., Ramanathan A., Ravichandran D., Zhu Y., Thippanna V., Sobczak M. T., Sajikumar A., Chambers L. B., Guo S., Kannan A. M., Song K. (2024). Machine Learning-Enabled
Direct Ink Writing of Conductive Polymer Composites for Enhanced Performance
in Thermal Management and Current Protection. Energy Storage Mater..

[57] Franklin R., Xu W., Ravichandran D., Jambhulkar S., Zhu Y., Song K. (2021). Reinforcing
Carbonized Polyacrylonitrile Fibers with Nanoscale Graphitic Interface-Layers. J. Mater. Sci. Technol..

[58] Xu W., Zhu Y., Ravichandran D., Jambhulkar S., Kakarla M., Bawareth M., Lanke S., Song K. (2021). Review of Fiber-Based Three-Dimensional
Printing for Applications Ranging from Nanoscale Nanoparticle Alignment
to Macroscale Patterning. ACS Appl. Nano Mater..

[59] Xu W., Jambhulkar S., Ravichandran D., Zhu Y., Kakarla M., Nian Q., Azeredo B., Chen X., Jin K., Vernon B., Lott D. G., Cornella J. L., Shefi O., Miquelard-Garnier G., Yang Y., Song K. (2021). 3D Printing-Enabled
Nanoparticle Alignment: A Review of Mechanisms and Applications. Small.

[60] Song K., Zhang Y., Meng J., Green E. C., Tajaddod N., Li H., Minus M. L. (2013). Structural
Polymer-Based Carbon Nanotube Composite
Fibers: Understanding the Processing-Structure-Performance Relationship. Materials.

[61] Zhang X., Liu T., Sreekumar T. V., Kumar S., Hu X., Smith K. (2004). Gel Spinning
of PVA/SWNT Composite Fiber. Polymer.

[62] Meng J., Zhang Y., Song K., Minus M. L. (2014). Forming Crystalline
Polymer-Nano Interphase Structures for High-Modulus and High-Tensile/Strength
Composite Fibers. Macromol. Mater. Eng..

[63] Song K., Zhang Y., Meng J., Minus M. L. (2013). Lubrication of Poly­(Vinyl
Alcohol) Chain Orientation by Carbon Nano-Chips in Composite Tapes. J. Appl. Polym. Sci..

[64] Minus M. L., Chae H. G., Kumar S. (2009). Interfacial Crystallization in Gel-Spun
Poly (Vinyl Alcohol)/Single-Wall Carbon Nanotube Composite Fibers. Macromol. Chem. Phys..

