# Supplementary material for: Multilayered Fabrication Containing Wind Turbine Blade Solid Wastes for High-Performance Composite Fibers
Source: ACS Mater Au. 2025 Jul 17;5(5):809–22. doi: 10.1021/acsmaterialsau.5c00041 (PMC12426776; doi:10.1021/acsmaterialsau.5c00041)
Supplement: Supplementary file 1 [file mg5c00041_si_001.pdf]

Multilayered fabrication Containing Wind Turbine Blade Solid Wastes for High-Performance Composites Fibers

Varunkumar Thippanna[1], Arunachalam Ramanathan[1], Dhanush Patil[1], M. Taylor Sobczak[1], T aylor G Theobald[1], Sri Vaishnavi Thummalapalli[1], Xiao Sun[2], Churan Yu[1], Ian Doran[1], Chao Sui[1], Joshua Were[1], Xianqiao Wang[3], Sui Yang[4], Xin Xu[5], Arunachala Nadar Mada Kannan[5], Amir Asadi[6], Ayman Nafady[7], Abdullah M Al-Enizi[7], Mohammad K Hassan[8], Kenan Song[9]*

[1]Mechanical Engineering, College of Engineering, University of Georgia, 302 E Campus Rd, Athens, GA, 30602, United States.

[2]Department of Mechanical and Industrial Engineering, College of Engineering, Northeastern University, 360 Huntington Ave, Boston, MA, 02115, United States.

[3]School of ECAM, College of Engineering, University of Georgia, Athens, GA, 30602, United States.

[4]Materials Science and Engineering, School for Engineering of Matter, Transport and Energy (SEMTE), Ira A. Fulton Schools of Engineering, Arizona State University, Tempe, AZ, 85281, United States.

[5]The Polytechnic School (TPS), Ira Fulton Schools of Engineering, Arizona State University, Mesa, AZ, 85212, United States.

[6]Manufacturing and Mechanical Engineering Technology, Department of Engineering Technology and Industrial Distribution, Texas A&M University, College Station, TX, 77843-3367, United States.

[7]Department of Chemistry, College of Science, King Saud University, Riyadh, 11451, Saudi Arabia.

[8]Center for Advanced Materials, Qatar University, Doha, 2713, Qatar.

[9]*Associate Professor, Mechanical Engineering, College of Engineering, University of Georgia (UGA), 302 E. Campus Rd., Athens, GA, 30602, United States.

*Corresponding author, Email: kenan.song@uga.edu

Number of Pages: 18

Number of Figures: 12

Number of Tables: 3

Table of Contents

1. Fiber spinning and draw-ability conditions 5

2. Mechanical analysis of precursor fibers 8

3. Fiber morphologies for precursor fibers 9

4. TGA for precursor composite fibers 10

5. Heat treatment process and reactions 11

6. Fiber morphologies of Carbonized fibers 12

7. TGA of Carbonized Fibers and Polymer-CF 13

8. Thermal stability of precursor fiber 14

9. Polymer under the influence of Carbonized fiber 15

10. Electrical properties of fibers 16

11. Thermogravimetric analysis (TGA) for Mechanically Recycled Wind Turbine Blades 17

12. References 18

Table of Figures

Figure S1. (a_1) Custom-designed multi-layered spinneret (a_2) The solution was injected into an air gap of 1.5–2.0 cm before entering the coagulation bath (a_3) Continuous collection of the As-spun fibers (a_4) Drawn fibers with decreased fiber diameter (a_5) Slower winder with the previously collected fibers (a_6) Drawing process with both the winders at different speeds, (a_7) High DR fibers. 5

Figure S2. Mechanical properties of the precursor fibers with different layers. (a_1) Stress-strain graph for precursor 32-layered fibers. (a_2) 64-layered fibers. (a_3) 128-layered fibers. (a_4) 256-layered fibers. 8

Figure S3. Morphologies of 256-layered fibers with different GF wt.% 9

Figure S4. Heat treatment reactions of PAN fibers during the stabilization and carbonization process.... 11

Figure S5. The heat treatment process of precursors with stabilization in the air atmosphere and carbonization in an inert atmosphere (N_2) to produce carbonized fibers. 11

Figure S6. Morphologies of 256 layered carbonized fibers. 12

Figure S7. TGA of the carbonized fibers and polymer-CF to determine the composite fiber's degradation temperature and residual wt.%. 13

Figure S8. Vertical burn test ASTM D6413 for precursor fibers. 14

Figure S9. Polymer behavior under the influence of carbonized fibers. 15

Figure S10. Laboratory set-up to determine the electrical conductivity of CF and polymer composites. . 16

Figure S11. Resistivity with increasing fiber numbers of carbonized fibers and polymer composites 16

Figure S12. TGA of wind turbine blade-based solid waste to determine the glass fiber concentration 17

Table of Tables

Table S1. Fiber increasing draw ratios of stretched fibers before breaking. ... 6

Table S2. Degradation temperatures and final residues of PAN and PAN-GF composite fibers. ... 10

Table S3. Fiber type and their electrical properties ... 16

1. Fiber spinning and draw-ability conditions

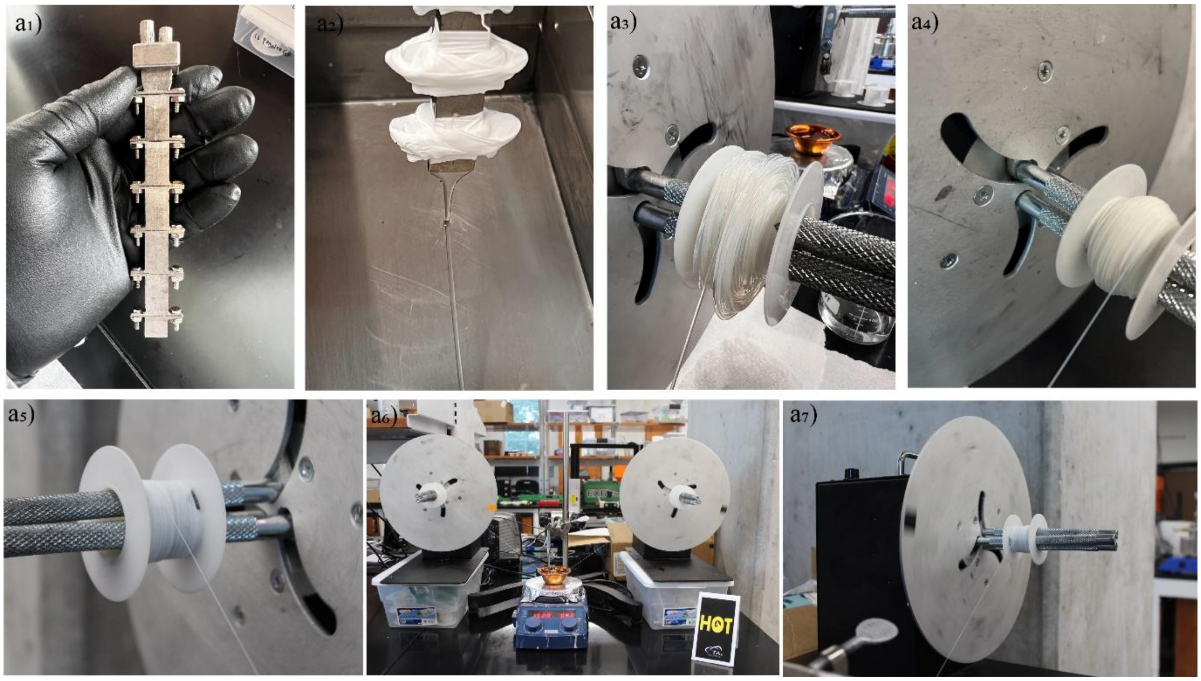

Figure S1. (a_1) Custom-designed multi-layered spinneret (a_2) The solution was injected into an air gap of 1.5–2.0 cm before entering the coagulation bath (a_3) Continuous collection of the As-spun fibers (a_4) Drawn fibers with decreased fiber diameter (a_5) Slower winder with the previously collected fibers (a_6) Drawing process with both the winders at different speeds, (a_7) High DR fibers.

Table S1. Fiber increasing draw ratios of stretched fibers before breaking.

	Fiber type	Spinning medium	Max speed (sec)	Min speed (sec)	Individual draw ratio	Total Draw ratio
32 Layered fibers	10PAN	Water (85ºC)	2.86	1.35	2.12	2.12
			3.16	1.18	2.68	5.68
			3.70	2.02	1.83	10.40
		Oil (125ºC)	3.16	1.70	1.85	19.24
		Oil (135ºC)	2.50	1.61	1.55	29.81
		Oil (145ºC)	4.18	3.21	1.30	**38.76**
	10PAN-1GF	Water (85ºC)	3.04	1.26	2.41	2.41
			2.90	1.60	1.81	4.37
			2.77	1.06	2.61	11.38
		Oil (125ºC)	2.99	1.55	1.93	21.97
		Oil (135ºC)	3.10	1.89	1.64	36.04
		Oil (145ºC)	3.42	2.50	1.37	**49.37**
	10PAN-2GF	Water (85ºC)	2.83	1.47	1.93	1.93
			3.24	0.99	3.27	6.31
			2.91	1.87	1.56	9.84
		Oil (125ºC)	3.52	2.72	1.30	12.79
		Oil (135ºC)	4.50	1.83	2.46	31.46
		Oil (145ºC)	3.11	2.19	1.42	44.69
			4.78	3.67	1.30	**58.21**
	10PAN-4GF	Water (85ºC)	2.80	1.45	1.93	1.93
			4.18	1.96	2.13	4.11
			2.90	1.64	1.77	7.28
		Oil (125ºC)	3.74	1.70	2.20	16.01
		Oil (135ºC)	3.75	1.77	2.12	33.92
		Oil (145ºC)	4.03	2.37	1.70	**57.68**
64 Layered fibers	10PAN	Water (85ºC)	3.00	1.57	1.91	1.91
			3.40	1.54	2.21	4.22
			2.80	1.36	2.06	8.69
		Oil (125ºC)	2.48	1.39	1.78	15.48
		Oil (135ºC)	3.21	1.82	1.76	27.24
		Oil (145ºC)	2.80	1.83	1.53	**41.68**
	10PAN-1GF	Water (85ºC)	3.03	1.41	2.15	2.15
			4.11	1.72	2.40	5.16
			2.93	2.00	1.47	7.59
		Oil (125ºC)	3.90	2.01	1.94	14.72
		Oil (135ºC)	6.02	2.88	2.09	30.75
		Oil (145ºC)	4.60	3.12	1.47	**45.21**
	10PAN-2GF	Water (85ºC)	2.46	1.19	2.07	2.07
			3.11	1.63	1.90	3.93
			3.00	1.85	1.62	6.37
		Oil (125ºC)	4.03	1.68	2.40	15.29
		Oil (135ºC)	2.45	1.67	1.47	22.48
		Oil (145ºC)	3.88	2.67	1.45	32.59
			3.32	2.38	1.40	**45.63**
	10PAN-4GF	Water (85ºC)	3.21	1.23	2.61	2.61
			2.70	1.29	2.10	5.48
			4.60	2.05	2.24	12.28
		Oil (125ºC)	2.98	1.81	1.65	20.26
		Oil (135ºC)	3.93	2.41	1.63	33.02
		Oil (145ºC)	3.16	2.15	1.47	**48.54**
128 Layered fibers	10PAN	Water (85ºC)	2.92	1.09	2.67	2.67
			3.75	1.06	3.54	9.42
			2.60	1.76	1.47	13.85
		Oil (125ºC)	3.17	1.68	1.88	26.05
		Oil (135ºC)	2.92	2.01	1.45	37.77
		Oil (145ºC)	2.44	2.04	1.20	**45.32**
	10PAN-1GF	Water (85ºC)	2.43	1.06	2.30	2.30
			2.52	1.29	1.95	4.49
			3.29	1.50	2.19	9.85
		Oil (125ºC)	3.25	1.38	2.35	23.15
		Oil (135ºC)	2.81	1.99	1.41	32.71
		Oil (145ºC)	3.66	2.65	1.38	**45.14**
	10PAN-2GF	Water (85ºC)	2.87	1.54	1.86	1.86
			2.99	1.61	1.86	3.46

			2.71	1.60	1.69	5.85
		Oil (125ºC)	3.77	1.74	2.16	12.63
		Oil (135ºC)	3.44	1.68	2.05	25.89
		Oil (145ºC)	3.54	2.17	1.63	**42.20**
	10PAN-4GF	Water (85ºC)	2.69	1.49	1.80	1.80
			2.46	1.11	2.22	4.00
			2.80	1.71	2.23	8.91
		Oil (125ºC)	2.78	1.68	1.65	14.70
		Oil (135ºC)	3.14	1.61	1.95	28.67
		Oil (145ºC)	3.53	2.08	1.70	**48.74**
256 Layered fibers	10PAN	Water (85ºC)	3.93	1.79	2.20	2.20
			3.89	1.51	2.58	5.68
			2.87	2.06	1.40	7.89
		Oil (125ºC)	3.53	1.86	1.89	14.91
		Oil (135ºC)	2.96	1.80	1.64	24.45
		Oil (145ºC)	4.36	2.51	1.73	**42.31**
	10PAN-1GF	Water (85ºC)	2.70	1.11	2.43	2.43
			2.56	1.59	1.61	3.91
			2.88	1.49	1.93	7.55
		Oil (125ºC)	2.91	1.66	1.75	13.21
		Oil (135ºC)	3.80	2.40	1.58	20.88
		Oil (145ºC)	3.83	2.26	1.69	**35.28**
	10PAN-2GF	Water (85ºC)	3.69	1.76	2.09	2.09
			3.20	1.85	1.73	3.62
			3.01	1.87	1.60	5.79
		Oil (125ºC)	2.91	1.35	2.15	12.44
		Oil (135ºC)	3.22	1.92	1.67	20.77
		Oil (145ºC)	3.01	1.61	1.88	**39.00**
	10PAN-4GF	Water (85ºC)	3.20	1.35	2.37	2.37
			4.11	1.99	2.06	4.89
			3.31	2.04	1.62	7.91
		Oil (125ºC)	3.86	2.16	1.78	14.08
		Oil (135ºC)	4.27	2.41	1.77	24.92
		Oil (145ºC)	4.06	2.89	1.40	**34.89**

91

2. Mechanical analysis of precursor fibers

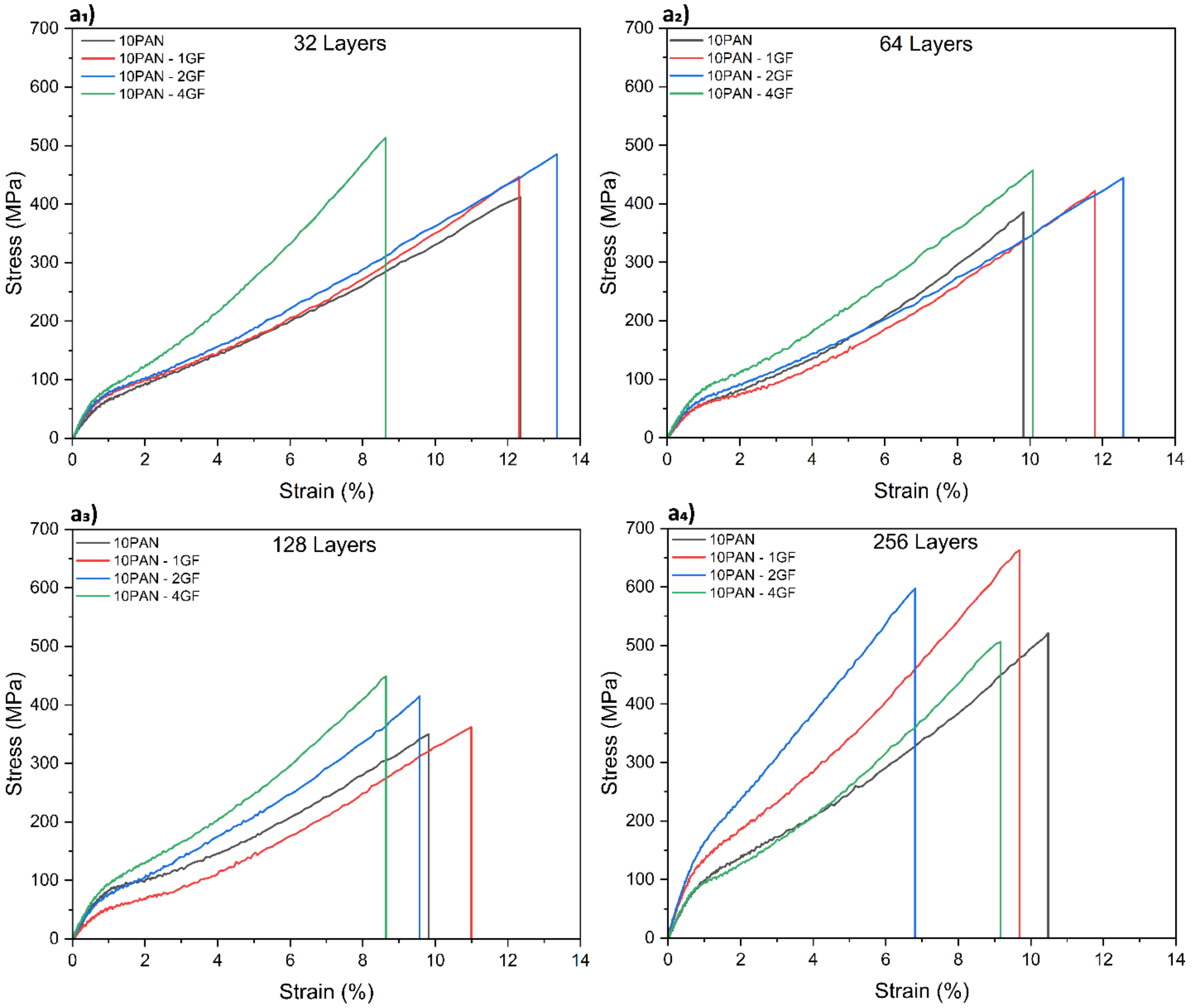

Figure S2. Mechanical properties of the precursor fibers with different layers. (a_1) Stress-strain graph for precursor 32-layered fibers. (a_2) 64-layered fibers. (a_3) 128-layered fibers. (a_4) 256-layered fibers.

3. Fiber morphologies for precursor fibers

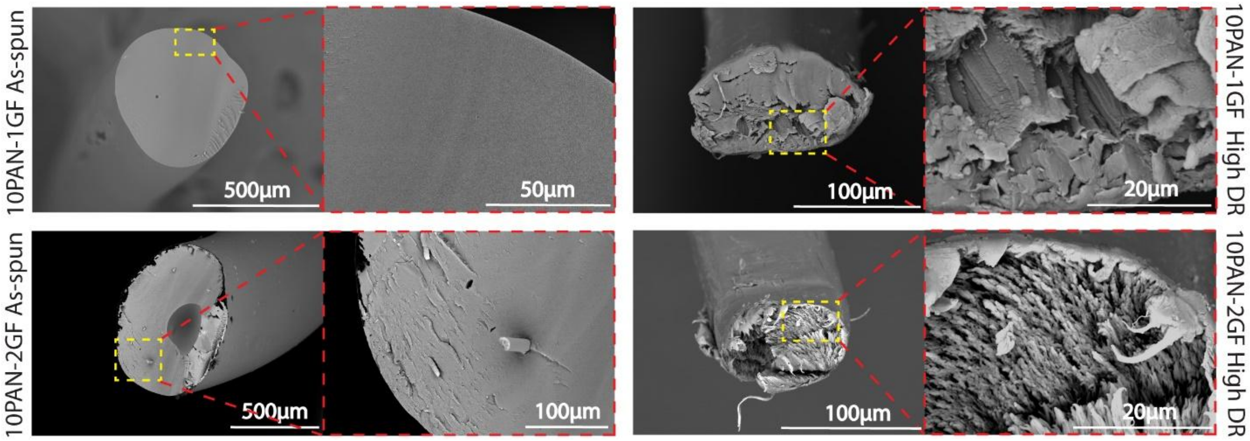

Figure S3. Morphologies of 256-layered fibers with different GF wt.%

4. TGA for precursor composite fibers

Table S2. Degradation temperatures and final residues of PAN and PAN-GF composite fibers.

Layers produced	Fiber type	Offset (Initial degradation) temperature (°C)	Endset (Final degradation) temperature (°C)	Residual weight at 900 °C (wt.%)
32	10PAN	287.76	435.61	36.69
	10PAN-1GF	289.42	440.54	34.96
	10PAN-2GF	288.33	440.05	32.67
	10PAN-4GF	287.59	444.35	35.17
64	10PAN	287.33	444.77	36.74
	10PAN-1GF	290.87	444.71	38.86
	10PAN-2GF	289.63	443.45	37.77
	10PAN-4GF	290.62	447.47	39.50
128	10PAN	290.15	443.44	34.43
	10PAN-1GF	285.65	447.06	35.86
	10PAN-2GF	288.48	439.84	30.61
	10PAN-4GF	291.60	442.07	39.30
256	10PAN	290.16	437.49	37.70
	10PAN-1GF	288.14	438.52	35.73
	10PAN-2GF	293.45	439.65	38.91
	10PAN-4GF	289.83	437.98	41.23

5. Heat treatment process and reactions

The stabilization process is significantly influenced by the heat generated during the reaction. Dehydrogenation, a process in which hydrogen is removed from the fiber, often as water in the presence of oxygen, can occur either before or after cyclization, which is an exothermic reaction. This dehydrogenation forms a double bond between carbon atoms, stabilizing the carbon backbone. Cyclization occurs when the nitrile group (C≡N) reacts, forming double bonds and creating a fused pyridine ring structure.[1,2]

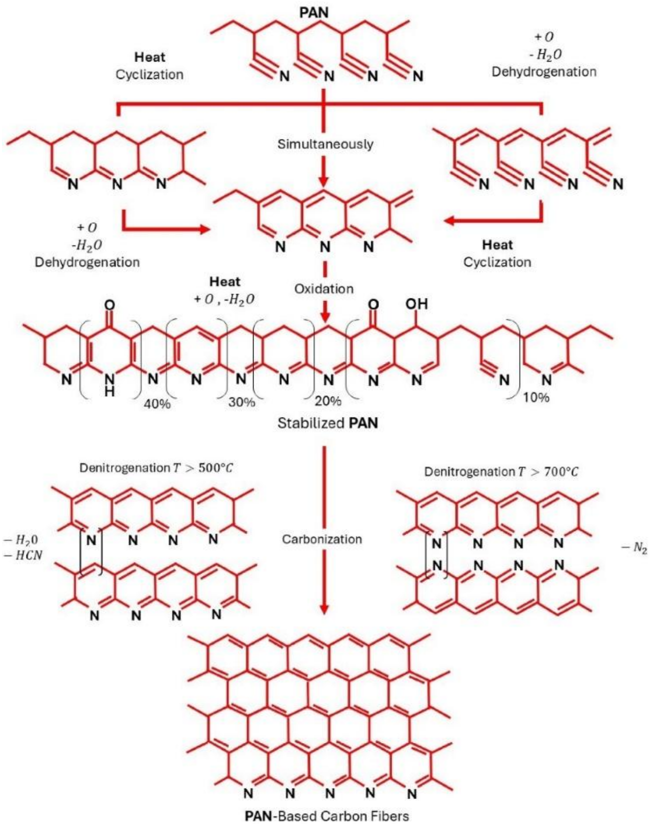

Figure S4. Heat treatment reactions of PAN fibers during the stabilization and carbonization process.

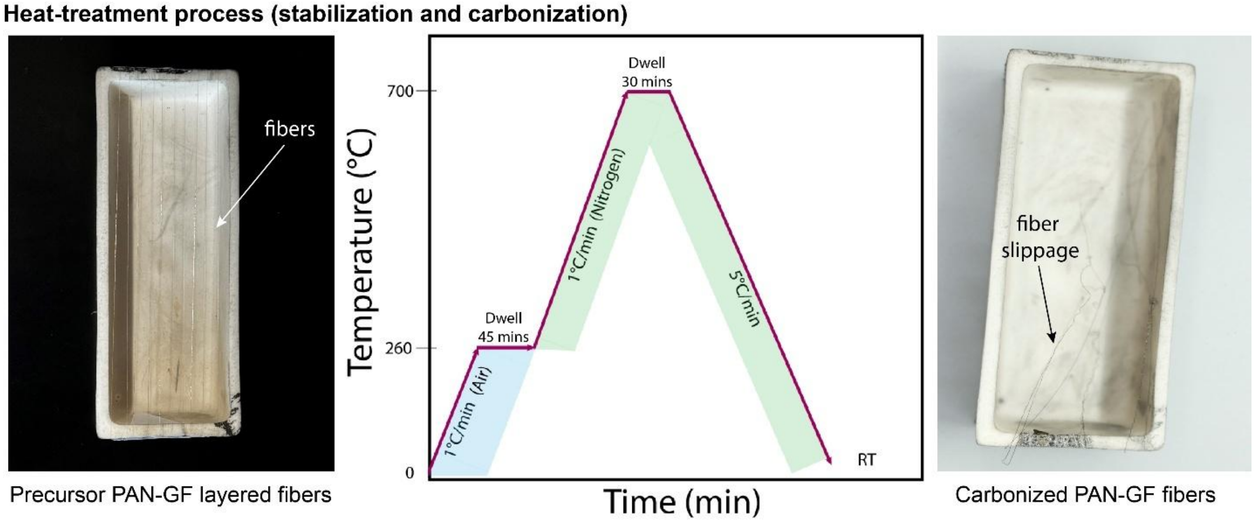

Figure S5. The heat treatment process of precursors with stabilization in the air atmosphere and carbonization in an inert atmosphere (N_2) to produce carbonized fibers.

6. Fiber morphologies of Carbonized fibers

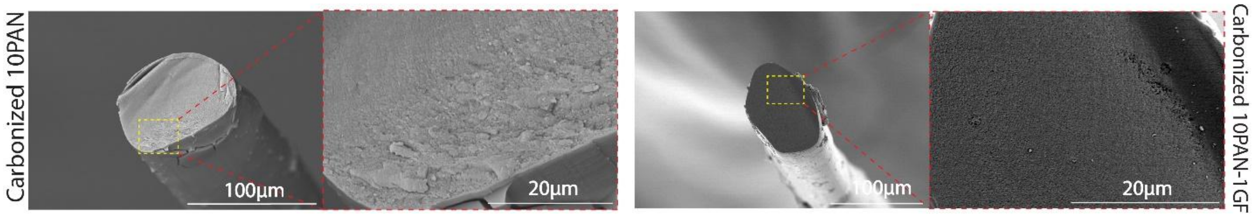

Figure S6. Morphologies of 256 layered carbonized fibers.

7. TGA of Carbonized Fibers and Polymer-CF

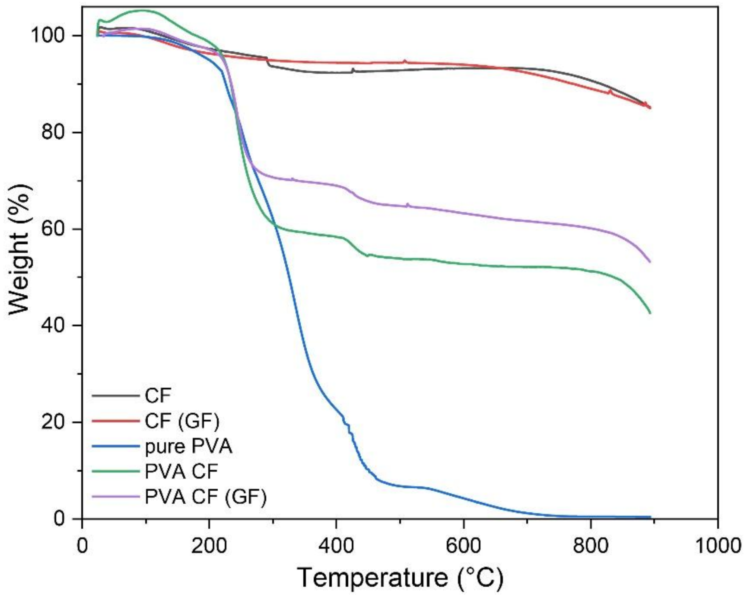

Figure S7. TGA of the carbonized fibers and polymer-CF to determine the composite fiber's degradation temperature and residual wt.%.

8. Thermal stability of precursor fiber

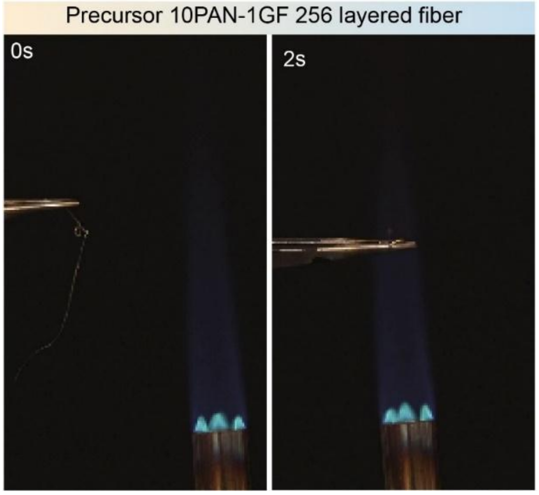

Figure S8. Vertical burn test ASTM D6413 for precursor fibers.

9. Polymer under the influence of Carbonized fiber

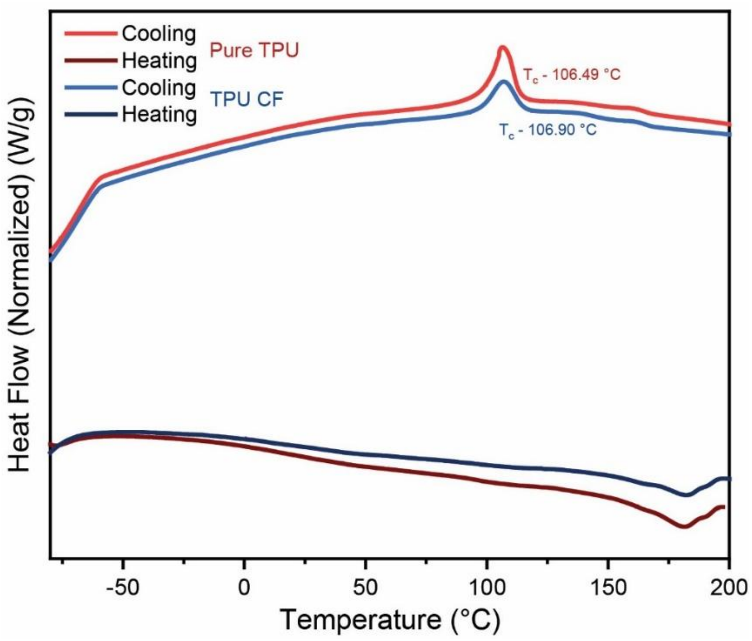

Figure S9. Polymer behavior under the influence of carbonized fibers.

10. Electrical properties of fibers

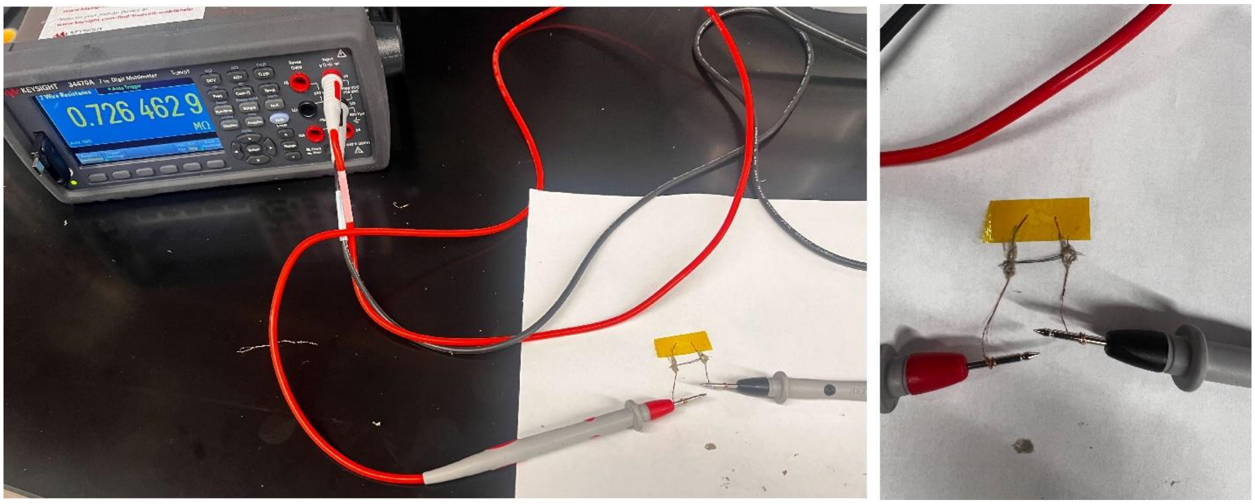

Figure S10. Laboratory set-up to determine the electrical conductivity of CF and polymer composites.

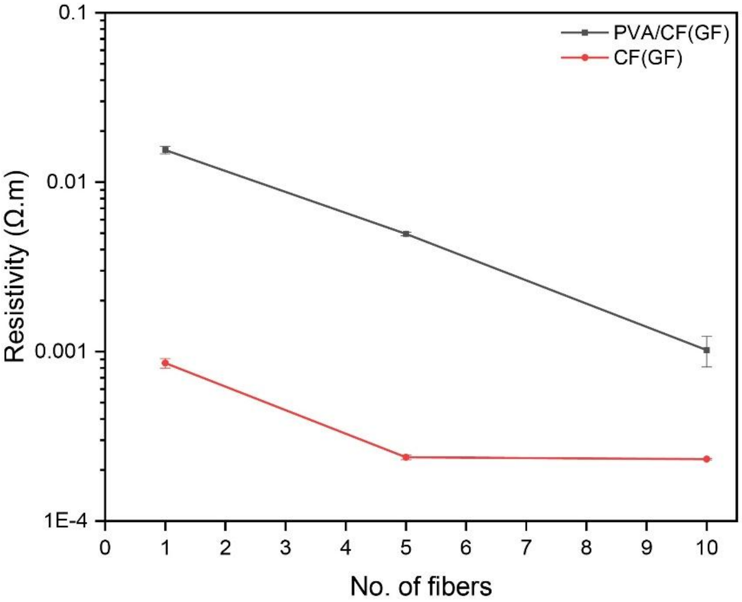

Figure S11. Resistivity with increasing fiber numbers of carbonized fibers and polymer composites

Table S3. Fiber type and their electrical properties

Fiber type	No. of fibers	Resistance (MΩ)	Resistivity (Ω·m)	Conductivity (S/m)
PVA/CF(GF)	1	58.54 ± 2.97	1.54e-2 ± 7.85e-4	6.47e-5 ± 3.24e-6
	5	18.72 ± 0.44	4.95e-3 ± 1.17e-4	2.02e-4 ± 4.84e-6
	10	3.86 ± 0.78	1.02e-2 ± 2.08e-4	1.01e-3 ± 1.69e-4
CF(GF)	1	2.64 ± 0.17	8.55e-4 ± 5.64e-5	1.17e-3 ± 7.64e-5
	5	0.73 ± 0.02	2.37e-4 ± 7.84e-6	4.21e-3 ± 1.43e-4
	10	0.71 ± 0.01	2.31e-4 ± 2.31e-6	4.32e-3 ± 4.33e-5

11. Thermogravimetric analysis (TGA) for Mechanically Recycled Wind Turbine Blades

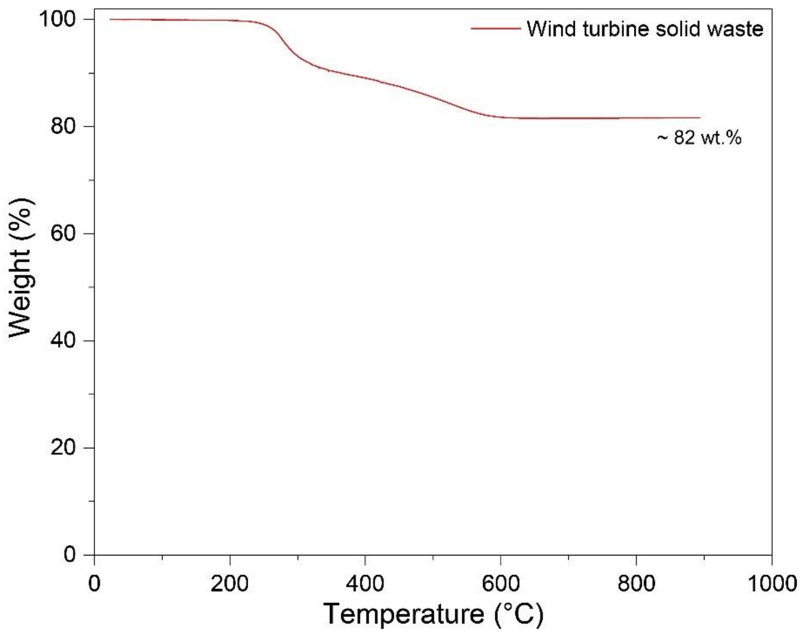

Figure S12. TGA of wind turbine blade-based solid waste to determine the glass fiber concentration

12. References

(1) Hameed, N.; Sharp, J.; Nunna, S.; Creighton, C.; Magniez, K.; Jyotishkumar, P.; Salim, N. V.; Fox, B. Structural Transformation of Polyacrylonitrile Fibers during Stabilization and Low Temperature Carbonization. *Polym Degrad Stab* **2016**, *128*, 39–45.

(2) Konstantopoulos, G.; Soulis, S.; Dragatogiannis, D.; Charitidis, C. Introduction of a Methodology to Enhance the Stabilization Process of PAN Fibers by Modeling and Advanced Characterization. *Materials 2020, Vol. 13, Page 2749* **2020**, *13* (12), 2749.
